# ETS Variant Transcription Factor 6 Promotes Glucose Metabolism Reprogramming in HCC

**DOI:** 10.1111/jcmm.71029

**Published:** 2026-02-03

**Authors:** Chunmei Guo, Lingqian Xie, Huiqing Yin, Lina Yi, Lin Jin, Xiangwei Liu, Qingqing Zhang, Zijian Li, Shuqing Liu, Ming‐Zhong Sun

**Affiliations:** ^1^ Department of Biotechnology, College of Basic Medical Sciences Dalian Medical University Dalian Liaoning China; ^2^ Department of Biochemistry, College of Basic Medical Sciences Dalian Medical University Dalian Liaoning China; ^3^ Department of Pathology and Forensic Medicine, College of Basic Medical Sciences Dalian Medical University Dalian Liaoning China; ^4^ Liaoning Key Laboratory of Cancer Stem Cell Research, College of Basic Medical Sciences Dalian Medical University Dalian Liaoning China

**Keywords:** CRKL, ETV6, glucose metabolism reprogramming, glycogen shunt, miR‐429, PI3K/AKT pathway

## Abstract

Glucose metabolic reprogramming is a key hallmark of tumour cells, and the designed inhibitors targeting tumour glucose metabolism reprogramming may serve as an effective therapeutic strategy. The ETS Variant Transcription Factor 6 (ETV6) is a potent transcriptional repressor strongly associated with tumorgenesis. However, the precise role and underlying action mechanism of ETV6 in tumour glucose metabolism reprogramming remain unreported. In this study, we demonstrate that the ETV6‐miR‐429‐CRKL regulatory axis contributes to metabolism reprogramming in HCC. Overexpression or knockdown of ETV6 and CRKL enhances or inhibits the Warburg effect and glycogen synthesis in HCC cells both in vitro and in vivo. In contrast, miR‐429 overexpression and knockdown exert opposing effects on the Warburg effect compared to the overexpression and knockdown of ETV6 and CRKL. Moreover, miR‐429 regulates the rate of glycogen production and degradation by enhancing the activities of GCS and GPa to promote glycogen synthesis, subsequently coupling with the aerobic glycolytic pathway by mediating glycogen shunting. Mechanistically, ETV6 binds to the miR‐429 promoter, mediating glucose metabolic reprogramming in HCC cells by targeting CRKL via the PI3K/AKT pathway. Taken together, these findings reveal that the ETV6‐miR‐429‐CRKL regulatory circuitry plays a crucial role in glucose metabolic reprogramming in HCC, offering novel insight and a potential target for cancer therapy.

## 1 Introduction

Hepatocarcinoma (HCC) is the most common primary liver cancer and has the highest global incidence and mortality rates [1]. The combination of chemotherapy and surgical resection has significantly improved liver cancer treatment outcomes [2]. However, due to challenges in early diagnosis and the lack of effective monitoring and therapeutic targets, HCC patients often face poor prognoses [3]. Therefore, advancing HCC diagnosis and treatment relies on elucidating the molecular mechanisms underlying its development and progression. Identifying novel biomarkers and developing innovative diagnostic approaches for HCC progression, therapeutic strategies, and drug resistance will enable better patient management, diagnosis, and treatment.

Emerging as a novel hallmark of cancer, energy metabolism reprogramming is crucial for the proliferation, differentiation, and metastasis of tumour cells [4, 5]. The rapid proliferation and growth of solid tumours lead to a shortage of energy supply and oxygen deprivation. Glucose is the major energy source to support tumour growth and can also provide a carbon source for biosynthetic reactions. Accelerated glycolysis and increased cellular uptake of glucose through intricate metabolic reprogramming are frequently observed in human cancers [6, 7]. Cancer cells exhibit dramatic aberration of glucose metabolism; even in the presence of enough oxygen, tumour cells enhance glucose consumption with increased lactate production through aerobic glycolysis instead of oxidative phosphorylation for their growth, which is named as ‘Warburg effect’ [8, 9, 10]. Cancer cells preferentially select aerobic glycolysis over oxidative phosphorylation for glucose‐dependent ATP production due to mitochondrial impairments [11, 12]. Increased glycolysis benefits tumour cell growth and dissemination; metabolite lactate accumulation results in an acidic extracellular microenvironment causing the death of surrounding normal cells, ECM remodelling, promoting EMT and cancer cells metastasis [13, 14, 15]. Glycogen comprises a store of glucose and is mainly present in the liver and muscles; glycogen metabolism plays a critical role in a variety of tumours through glycogen‐related enzymes. Glycogen synthase (GCS) and glycogen phosphorylase a (GPa) catalyse the key steps of glycogen synthesis and breakdown [16]. Understanding the mechanisms of glucose metabolism can aid in developing new therapeutic targets and strategies for human cancers.

As a member of the E‐Twenty Six (ETS) transcription factor family, ETS variant 6 (ETV6) functions primarily as a transcriptional repressor involved in maintaining the vascular network, embryogenesis and haematogenesis [17, 18]. The *ETV6* gene is located on chromosome 12, spanning 240 kb and consisting of 8 exons. The encoded protein is modular, featuring a C‐terminal ETS DNA‐binding domain (encoded by exons 6–8) and an N‐terminal helix–loop–helix (HLH) domain (encoded by exons 3 and 4) [19, 20]. The ETS domain enables specific binding to purine‐rich DNA regions, while the HLH domain mediates homopolymerization and frequently induces constitutive activation of tyrosine kinase activity [21, 22]. ETV6 has emerged as a major oncogene with the ability to form fusion partners with numerous other genes [23, 24, 25, 26, 27]. Additionally, ETV6 missense mutations and translocations are frequently detected in cancers, and its dysregulation contributes to cancer development and progression [28, 29, 30, 31]. However, the biological function and regulatory mechanism of ETV6 in metabolism remain unclear. CRKL (CRK‐like), a member of the CRK (v‐crk sarcoma virus CT10 oncogene homologue) adapter protein family, is associated with the development and progression of various cancers. Our previous studies revealed that both CRKL and ETV6 dysregulation significantly influence the metastatic potential of HCC cells; moreover, ETV6 directly binds to CRKL and positively regulates its expression [32]. Furthermore, CRKL also regulates the glucose metabolism of HCC cells via the PI3K/AKT pathway by directly interacting with PI3K [33]. Based on these findings, we hypothesise that ETV6 may play an important role in regulating glucose metabolism in HCC cells.

The aim of this study was to probe the association of ETV6 with glucose metabolism and to explore its potential functional regulatory mechanism. We observed that ETV6 overexpression facilitated the Warburg effect and glycogen synthesis in HCC cells, whereas ETV6 knockdown inhibited these metabolic processes. Further analysis revealed that ETV6 interacts with miR‐429 to mediate glucose metabolic reprogramming in HCC cells by targeting CRKL. Mechanistically, the ETV6‐miR‐429‐CRKL regulatory axis modulates glucose metabolism in HCC cells through the PI3K/AKT pathway. Interestingly, miR‐429 was found to inhibit the Warburg effect while promoting glycogen synthesis in HCC cells; additionally, miR‐429 is coupled to the aerobic glycolysis pathway by mediating the glycogen shunt. Our findings suggest that the ETV6‐miR‐429‐CRKL regulatory loop could serve as a promising metabolism target for antitumor therapy.

## 2 Material and Methods

### 2.1 Cell Lines and Cell Culture

Human HCC HepG2 and HCCLM3 cells are routinely stored in liquid nitrogen in our laboratory. They are cultured in DMEM medium (Gibco, USA) supplemented with 15% fetal bovine serum (FBS, TransGen, China) and 100 U/mL penicillin–streptomycin (Gibco, USA), and maintained in a humidified incubator at 37°C with 5% CO_2_.

### 2.2 Small Interfering RNA (siRNA) Design, Plasmid Construction and Transient Transfection

For the knockdown of ETV6 and CRKL, specific siRNAs were designed based on the ETV6 sequence (Genbank: NM_001987.4; siETV6‐1: 5′‐CAAUAUAGGUCUCAGAAAUCC‐3′; siETV6‐2: 5′‐GCAUUAAGCAGGAACGAAU‐3′; siETV6‐3: 5′‐GGGAUUACGUCUAUCAGUU‐3′) and CRKL sequence (Genbank: NM_005207.3; siCRKL1: 5′‐CCUGGAAUAUGUACGGACUCUGUAU‐3′; siCRKL2: 5′‐GGGUUGGGAUGAUUCCUGUCCCUUA‐3′; siCRKL3: 5′‐CAUGGAAAUAGGAAUUCCAACAGUU‐3′) using Invitrogen, siDirect, and Whitehead software. Meanwhile, a non‐targeting siRNA with the sequence 5′‐UUCUCCGAACGUGUCACGUTT‐3′ was designed as a negative control (NC). The 6 μL siRNA mixtures (siETV6‐1: siETV6‐2: siETV6‐3:1:1:1, siCRKL‐1: siCRKL‐2: siCRKL‐3:1:1:1) were transiently transfected into HepG2 and HCCLM3 cells using 5 μL Lipofectamine 2000 (Invitrogen, USA) and incubated at 37°C with 5% CO_2_ for 48 h, respectively.

For ETV6 and CRKL overexpression, the full‐length coding sequence of *ETV6* or *CRKL* were amplificated by RT‐PCR using the forward primer 5′‐TAGCTAGCG CCACCATGTCTGAGACTC CTGCTC‐3′ or 5′‐TATCTAGAGCCACCATGTCCTCCGCCAGGTT‐3′ and reverse primer 5′‐CGTTCGAAGGGCCTCGTTTTCATCTGGGTTT‐3′ or 5′‐GCCGCGCTT CGAATCAGCATTCATCTTCTTGG‐3′, the fragment was then cloned into the PCDH‐EF1‐MCS‐T2A‐Puro vector. The recombinant expression vectors PCDH‐EF1‐MCS‐T2A‐Puro‐ETV6 or PCDH‐EF1‐MCS‐T2A‐Puro‐CRKL (3 μg each) were transiently transfected into HepG2 and HCCLM3 cells using 5 μL of Lipofectamine 2000 (Invitrogen, USA), followed by incubation for 48 h at 37°C with 5% CO_2_.

For miR‐429 overexpression and knockdown, miR‐429 mimic, miR‐NC mimic, antagomiR‐429, and antagomiR‐NC inhibitor oligonucleotides were transiently transfected into HepG2 and HCCLM3 cells using 5 μL of Lipofectamine 2000 (Invitrogen, USA) and incubated for 48 h at 37°C with 5% CO_2_.

### 2.3 Quantitative Real‐Time PCR (qRT‐PCR) Assay

Total RNA was extracted from each group of cells and tissues using Trizol reagent (TransGen, China) and reverse transcribed using the EasyScript One‐Step gDNA Removal and cDNA Synthesis SuperMix kit (TransGen, China). Quantitative RT‐PCR was performed with TransStart Tip Green qPCR SuperMix (TransGen, China) on a StepOnePlus Real‐Time PCR system (Thermo Fisher, USA). snRNA U6 served as the internal reference and normalisation control for miR‐429. The relative expression levels of miR‐429 in different groups of cells and tissues were calculated using the 2^−ΔΔCT^ method.

### 2.4 Western Blotting (WB) Assay

Each group of cells and tissues was harvested and washed with PBS, followed by protein extraction using RIPA buffer. The lysate was centrifuged at 13406 *g* for 15 min at 4°C to collect the supernatant. Protein concentration was determined by the Bradford assay, and equal amounts of each sample were mixed with loading buffer, boiled for 5 min, and separated by 10% SDS‐PAGE. The resolved proteins were then transferred onto a nitrocellulose membrane, which was blocked with 5% (w/v) skim milk in TBST for 3 h at RT and incubated with primary antibodies overnight at 4°C. After incubation, the membrane was washed three times (10 min each) with TBST, followed by incubation with a horseradish peroxidase (HRP)‐conjugated secondary antibody for 3 h at RT. The membrane was then washed again three times (10 min each) with TBST. Protein bands were detected using an ECL substrate (Advansta, USA) and imaged using a Bio‐Rad ChemiDoc MP system (Bio‐Rad, 731BR00277, USA). GAPDH served as the internal control.

### 2.5 Glucose Oxidase‐Peroxidase (GOD‐POD) Assay

The effect of ETV6, CRKL and miR‐429 dysregulation on glucose uptake in HCC cells was assessed using a Glucose Detection Kit (Nanjing Jiancheng Bioengineering Institute). The culture medium from each group was collected in 48 h after transfection, transferred to a 0.6 mL Ep tube and centrifuged at 366 *g* for 10 min. Then, 2 μL of the supernatant was mixed with 200 μL of working solution in a 96‐well plate and incubated at 37°C for 15 min. The absorbances at 505 nm were measured using a microplate reader (Thermo). Meanwhile, for calibration, 2 μL of calibrator (5.55 mM) was used.

### 2.6 Lactate Oxidase (LOD) Assay

The effect of ETV6, CRKL, and miR‐429 deregulation on lactate production in HCC cells and tumour tissues was measured using a Lactate Detection Kit (Nanjing Jiancheng Bioengineering Institute). The cell culture medium from each group was collected in 48 h after transfection and tumour tissues were homogenised with saline on ice; then, the cell medium and homogenate were transferred to a 0.6 mL Ep tube and centrifuged at 566 *g* for 10 min. Next, 2 μL of supernatant was mixed with 20 μL chromogenic reagent and 100 μL enzyme working solution in a 96‐well plate, followed by incubation at 37°C for 10 min. Finally, 200 μL stop reagent was added to each well for absorbance assay at 530 nm using a microplate reader (Thermo).

### 2.7 pH Assay

The effect of ETV6, CRKL and miR‐429 deregulation on acidic extracellular microenvironment of HCC cells was measured using a pH meter. The medium of each group was collected 48 h after transfection, then the PHS‐3C pH meter was used to measure the pH of each group medium within 2 min.

### 2.8 Periodic Acid Schiff Reaction (PAS) Assay

The effect of ETV6, CRKL and miR‐429 dysregulation on glycogen synthesis in HCC cells was measured using a Glycogen Staining Kit (Solarbio). The cells of each group were collected 24 h after transfection, then the 100 μL cell suspension was loaded into a well of a 6‐well plate containing a 25 mm cover glass, followed by incubation at 37°C for 24 h. The cover glass was then washed with PBS, fixed with ice‐cold acetone, treated with periodic acid and rinsed with distilled water. Next, the cover glasses were incubated in 100 μL Schiff for 15 min, counterstained with haematoxylin, dehydrated using a graded ethanol series, cleared with xylene, and imaged by an upright microscope (Olympus, Japan) at 400×. Meanwhile, the paraffin‐embedded tumour tissue sections were dewaxed in xylene, rehydrated through a graded ethanol series, and processed by the Glycogen Staining Kit according to the manufacturer's protocol. Images were captured under an upright microscope (Olympus, Japan) at 200× magnification. The presence of amaranth‐coloured spherical particles in the cytoplasm indicated a PAS‐positive reaction.

### 2.9 Glycogen Content Assay

The effect of ETV6, CRKL, and miR‐429 deregulation on glycogen content of HCC cells and tumour tissues was measured using the CheKine Glycogen Assay Kit (Abbkine, USA) by anthrone method. The each group cells were collected in 48 h after transfection and sonication with 150 μL extraction buffer on ice for 30 × 3 s, then the homogenate was boiled for 20 min and the supernatant was collected by centrifugation at 5798 *g* for 10 min. Meanwhile, tumour tissues were hydrolyzed by lye. Then chromogen was added to each well for absorbance assay at 620 nm using a microplate reader (Thermo).

### 2.10 GCS And GPa Activity Assays

The GCS and GPa activities were measured using GCS and GPa Activity Assay Kit (Solarbio) by NADH and NADPH rate methods, respectively. The each group cells were collected in 48 h after transfection and sonication with 100 μL extraction buffer on ice for 3 min and the supernatant was collected by centrifugation at 5798 *g* for 10 min at 4°C. The absorbances at 340 nm were measured using a microplate reader (Thermo) according to the manufacturer's instructions.

### 2.11 Establishment of HCCLM3 Cell Line With Stable ETV6 and CRKL Knockdown

To investigate the impact of ETV6 and CRKL knockdown in vivo, specific shRNAs were designed based on the ETV6 and CRKL sequences: shETV6‐1: 5′‐GGAGGTCATACTGCATCAGAACTTCAAGAGAGTTCTGATGCAGTATGACCTCC‐3′; shETV6‐2:5′‐CCATAAGAACAGAACAAACATTTCAAGAGAATGTTTGTTCTGTTCTTATGG‐3′; shETV6‐3: 5′‐AGGAGCTGGATGAACAAATATTTCAAGAGAATATTTGTTCATCCAGCTCCT‐3′ and shCRKL1:5′‐ACTCGCGGGTCTCCCACTACATTCAAGAGATGTAGTGGGAGACCCGCGAGT‐3′; shCRKL2:5′‐GTGAGATCCTAGTGATAATAGTTCAAGAGACTATTATCACTAGGATCTCAC‐3′; shCRKL3: 5′‐GTCACAAGGATGAATATAATTCAAGAGATTATATTCATCCTTGTGAC‐3′, then inserted into the lentivirus expression vector pLenti‐U6‐shRNA‐CMV‐GFP‐2A‐Puro, the recombinant expression vectors pLenti‐U6‐shETV6‐CMV‐GFP‐2A‐Puro and pLenti‐U6‐shCRKL‐CMV‐GFP‐2A‐Puro were constructed and transfected into HCCLM3 cells, the HCCLM3 cells with ETV6 and CRKL stable knockdown were screened by 0.5 μg/mL puromycin for 21 days.

### 2.12 Construction of Nude Mice Xenograft Model

The nude mice xenograft model was employed to investigate the effects of ETV6 and CRKL3 knockdown or miR‐429 overexpression on the in vivo tumorigenicity and malignancy of HCCLM3 cells. 5 × 10^6^ cells from HCCLM3‐shETV6, HCCLM3‐shCRKL and HCCLM3‐shNC group were subcutaneously implanted into the right abdomen of BABL/c nude mouse (4–6 weeks, *n* = 5 per group). Meanwhile, 5 × 10^6^ wild‐type HCCLM3 cells were also subcutaneously implanted into the right abdomen of BABL/c nude mouse (4–6 weeks, *n* = 5 per group), after 10 days, when the tumours grew to a measurable size, 2.5 nM of NC‐agomir and miR‐429‐agomir in 50 μL PBS was injected into tumour on the abdomen of each BABL/c nude mouse every three days. Tumour size was measured and calculated using the formula: *V* = 1/2 × *a* × *b* ^2^ (*a* represents: length, *b* represents: width). On the 30th day after implantation, mice were euthanized, and primary tumour xenografts were excised, weighed, and stored appropriately. About 1/3 of each tumorous tissue was cut and frozen in liquid nitrogen for RNA and protein extraction or lactate production and glycogen content assays. The remaining tumorous tissues were fixed in 4% paraformaldehyde at RT for 24 h, paraffin‐embedded, sectioned into 3–5 μm slices for immunohistochemistry (IHC), haematoxylin–eosin (HE) and PAS staining assays.

### 2.13 HE Staining and IHC Assays

For HE staining, the paraffin sections were dewaxed using xylene, rehydrated through a graded ethanol series, and rinsed with distilled water. The sections were then stained with haematoxylin solution for 10 min, rinsed under running water for 5 min, differentiated in differentiation solution for 30 s, and immersed in water for 15 min. Subsequently, they were counterstained with eosin solution for 30 s. After staining, the sections were dehydrated in an ascending ethanol series, cleared in xylene, and mounted with neutral gum. Finally, the slides were examined and imaged under an optical microscope at 200× magnification.

For IHC assays of ETV6, CRKL, PI3K, p‐AKT, GLUT1, HKII, PFK1, PKM2, p‐GSK3β, and GSK3β, after dewaxing and rehydration as described above, the sections were immersed in 3% H_2_O_2_ for 10 min and then incubated with primary antibodies at 4°C overnight. Subsequently, the slides were treated with peroxidase‐conjugated streptavidin at 37°C for 20 min, washed with PBS, and stained with DAB at room temperature for 6 min. Finally, the slides were counterstained with haematoxylin, dehydrated through an ethanol gradient, cleared in xylene, and mounted with neutral gum. Imaging was performed using an optical microscope at 200× magnification. Brown staining indicated a positive reaction.

### 2.14 Data Processing and Statistical Analysis

The data are expressed as mean ± SD from at least three independent experiments. Differences between two groups were analysed using Student's *t*‐test. Statistical analyses were conducted with GraphPad Prism 6.0 software, and a *p*‐value < 0.05 was considered statistically significant.

## 3 Results

### 3.1 ETV6 Promotes Glycolysis of HCC Cells

**FIGURE 1:**
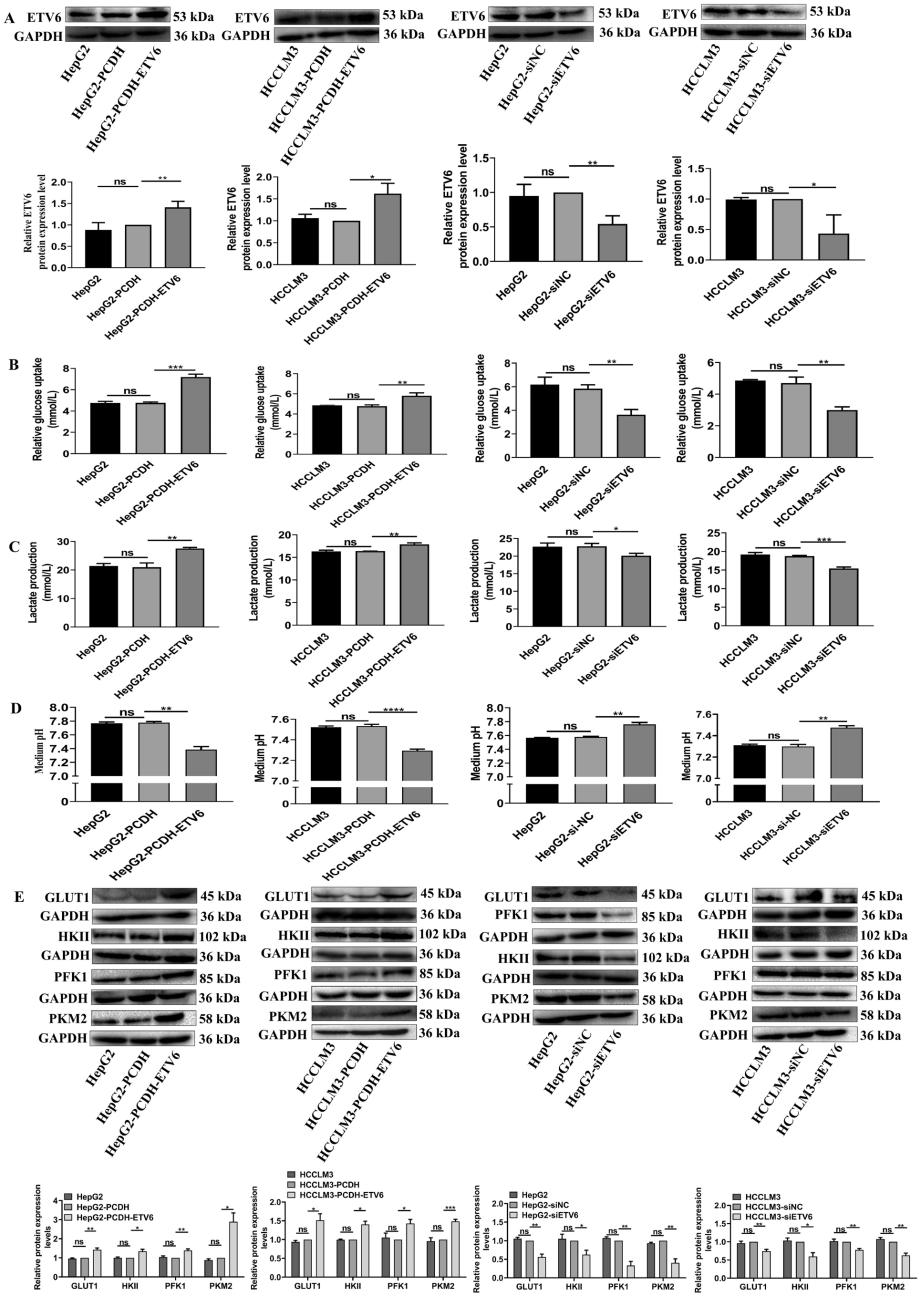
ETV6 promotes Warburg effect of HCC cells. (A) ETV6 was overexpressed or downregulated in HepG2 and HCCLM3 cells. (B) Glucose uptake was measured in HepG2 and HCCLM3 cells by GOD‐POD assay. (C) Lactate production was measured in HepG2 and HCCLM3 cells by LOD assay. (D) The pH value in culture medium was measured in HepG2 and HCCLM3 cells. (E) The protein expression levels of glycolysis‐related molecules were measured in HepG2 and HCCLM3 cells by WB assay. *, **, ***, ****Refer to *p* values < 0.05, 0.01, 0.001, 0.0001, ns refers to no statistical difference (*n* = 3).

To confirm the potential role of ETV6 in the regulation of HCC cell glycolysis, we overexpressed and knocked down ETV6 protein expression levels in HepG2 and HCCLM3 cells. ETV6 protein levels were increased by 41.0% (*p* = 0.0071) and 73.3% (*p* = 0.0419) in HepG2‐PCDH‐ETV6 and HCCLM3‐PCDH‐ETV6 cells compared with HepG2‐PCDH and HCCLM3‐PCDH cells, respectively, while there were no ETV6 protein expression level differences between HepG2 and HepG2‐PCDH or HCCLM3 and HCCLM3‐PCDH cells (Figure 1A). Meanwhile, in comparison with HepG2‐siNC and HCCLM3‐siNC cells, ETV6 protein expression levels decreased by 45.7% (*p* = 0.0026) and 56.7% (*p* = 0.0329) in HepG2‐siETV6 and HCCLM3‐siETV6 cells, respectively, while there were no ETV6 protein expression level differences between HepG2 and HepG2‐siNC or HCCLM3 and HCCLM3‐siNC cells (Figure 1A). The above results provide a control study for investigating the effect of ETV6 deregulation on glycolysis in HCC cells.

Glucose uptake, lactate production, cell medium pH assays were applied to measure the effect of ETV6 on HCC cell glycolysis. Firstly, GOD‐POD assay showed ETV6 enhanced glucose uptake of HCC cells. ETV6 overexpression increased the glucose uptake by 53.7% (*p* = 0.0001) and 21.9% (*p* = 0.0050) in both HepG2 and HCCLM3 cells (Figure 1B), in contrast, ETV6 silencing decreased the glucose uptake by 37.8% (*p* = 0.0022) and 36.4% (*p* = 0.0022) in both HepG2 and HCCLM3 cells (Figure 1B), while, there were no glucose consumption differences between HepG2 and HepG2‐PCDH, HCCLM3 and HCCLM3‐PCDH, HepG2 and HepG2‐siNC or HCCLM3 and HCCLM3‐siNC cells (Figure 1B).

Secondly, LOD assay showed ETV6 enhanced lactate production of HCC cells. The lactate production was increased by 31.5% (*p* = 0.0018) and 9.1% (*p* = 0.0012) after ETV6 overexpression in both HepG2 and HCCLM3 cells, while no lactate production differences were observed between HepG2 and HepG2‐PCDH or HCCLM3 and HCCLM3‐PCDH cells (Figure 1C). Meanwhile, ETV6 silencing decreased the lactate production by 11.8% (*p* = 0.0119) and 17.6% (*p* = 0.0002) in both HepG2 and HCCLM3 cells (Figure 1C), while there were no lactate production differences between HepG2 and HepG2‐siNC or HCCLM3 and HCCLM3‐siNC cells (Figure 1C). Lactate production results in an acidic extracellular microenvironment; pH assay showed ETV6 increased culture medium acidity. In comparison with HepG2‐PCDH and HCCLM3‐PCDH, the pH value was decreased in HepG2‐PCDH‐ETV6 and HCCLM3‐PCDH‐ETV6 cells culture medium (Figure 1D). While in comparison with HepG2‐siNC and HCCLM3‐siNC, the pH value was increased in HepG2‐siETV6 and HCCLM3‐siETV6 cells culture medium (Figure 1D).

Furthermore, we examined the protein expression levels of glycolysis‐related molecules. ETV6 overexpression increased the protein expression levels of GLUT1, HKII, PFK1, and PKM2 by 43.1% (*p* = 0.0072), 51.3% (*p* = 0.0415), 34.3% (*p* = 0.0296), and 40.0% (*p* = 0.0108), 38.7% (*p* = 0.0088), and 42.7% (*p* = 0.0174), 189.3% (*p* = 0.0149), and 48.7% (*p* = 0.0007) in HepG2 and HCCLM3 cells, respectively (Figure 1E). Meanwhile, ETV6 silencing decreased the protein expression levels of GLUT1, HKII, PFK1, and PKM2 by 44.0% (*p* = 0.0054), 26.0% (*p* = 0.0075), 37.7% (*p* = 0.0364), and 59.7% (*p* = 0.0176), 66.7% (*p* = 0.0036), and 22.0% (*p* = 0.0037), 59.3% (*p* = 0.0049), and 37.3% (*p* = 0.0043) in HepG2 and HCCLM3 cells, respectively (Figure 1E). In summary, the above results provide strong evidence that ETV6 enhances the Warburg effect in HCC cells.

### 3.2 ETV6 Promotes Glycogen Synthesis of HCC Cells

**FIGURE 2:**
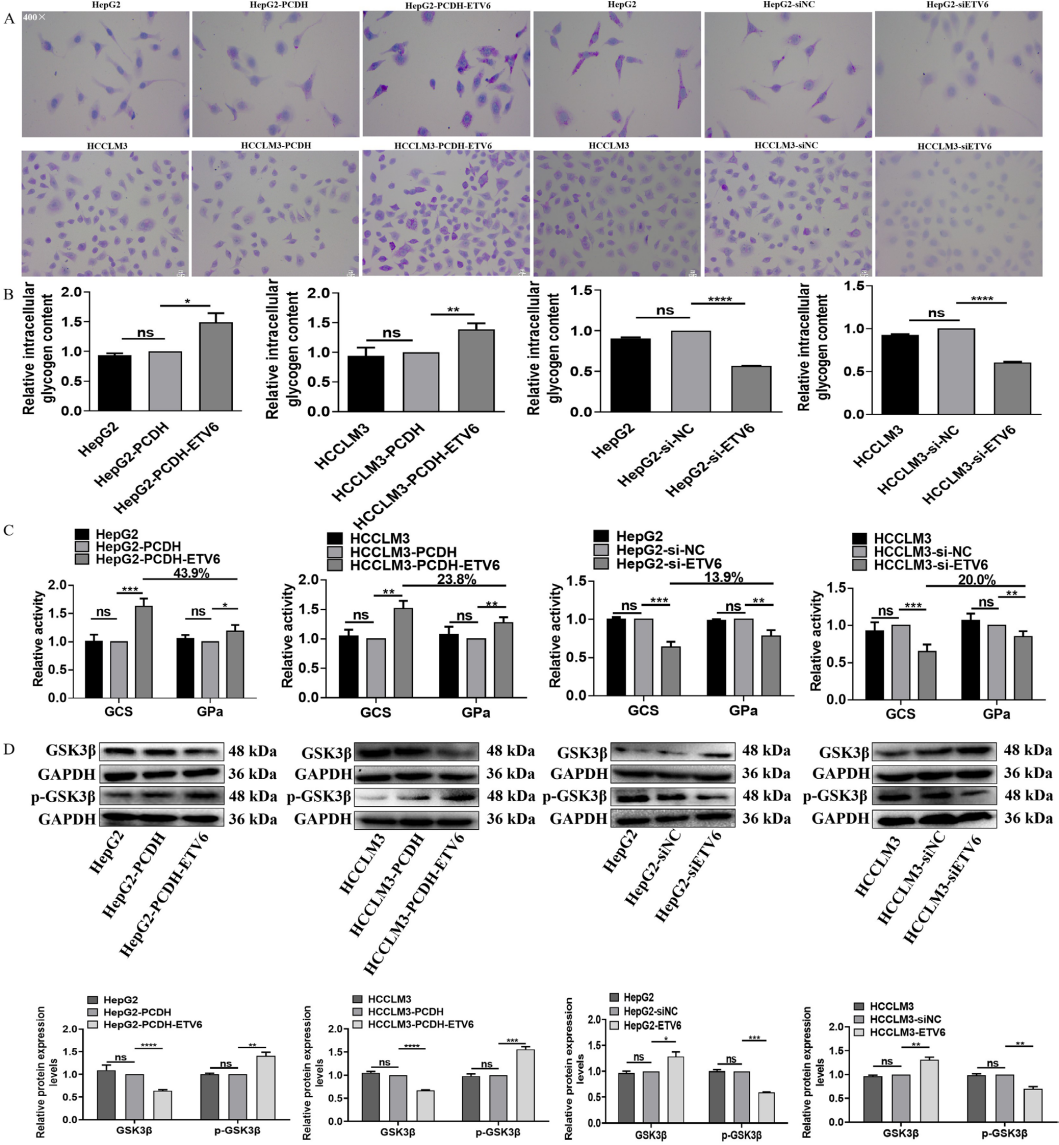
ETV6 promotes glycogen synthesis of HCC cells. (A) Glycogen synthesis was measured in HepG2 and HCCLM3 cells by PAS assay. (B) Intracellular glycogen content was measured in HepG2 and HCCLM3 cells by anthrone assay. (C) The GCS and GPa activities were measured in HepG2 and HCCLM3 cells by NADH and NADPH assays. (D) The protein expression levels of glycogen metabolism‐related molecules were measured in HepG2 and HCCLM3 cells by WB assay. *, **, ***, ****Refer to *p* values < 0.05, 0.01, 0.001, 0.0001, ns refers to no statistical difference (*n* = 3).

Glycogen synthesis is another important indicator of glucose metabolism. Glycogen synthesis, intracellular glycogen content and GCS and GPa activities assays were applied to measure the effect of ETV6 on HCC cell glycogen synthesis. Firstly, PAS assay showed ETV6 promoted glycogen synthesis of HCC cells. Compared to HepG2‐PCDH and HCCLM3‐PCDH, HepG2‐PCDH‐ETV6 and HCCLM3‐PCDH‐ETV6 cells exhibited strong positive PAS reaction with amaranth spherical particle glycogen distributed in cytoplasm (Figure 2A). In contrast, compared to HepG2‐siNC and HCCLM3‐siNC, HepG2‐siETV6 and HCCLM3‐siETV6 cells exhibited weak positive PAS reaction with amaranth spherical particle glycogen reduced in cytoplasm (Figure 2A).

Secondly, anthrone assay showed ETV6 increased the intracellular glycogen content of HCC cells. ETV6 overexpression increased the intracellular glycogen content by 48.9% (*p* = 0.0348) and 38.9% (*p* = 0.0025) in both HepG2 and HCCLM3 cells (Figure 2B), in contrast, ETV6 silencing decreased the intracellular glycogen content by 43.1% (*p* < 0.00001) and 39.5% (*p* < 0.0001) in both HepG2 and HCCLM3 cells (Figure 2B), while, there were no intracellular glycogen content differences between HepG2 and HepG2‐PCDH, HCCLM3 and HCCLM3‐PCDH, HepG2 and HepG2‐siNC or HCCLM3 and HCCLM3‐siNC cells (Figure 2B).

Thirdly, NADH and NADPH assays showed ETV6 enhanced the GCS and GPa activities of HCC cells. Compared to HepG2‐PCDH and HCCLM3‐PCDH, the GCS activity of HepG2‐PCDH‐ETV6 and HCCLM3‐PCDH‐ETV6 cells was enhanced by 62.6% (*p* = 0.0001) and 51.4% (*p* = 0.0005), the GPa activity of HepG2‐PCDH‐ETV6 and HCCLM3‐PCDH‐ETV6 cells was enhanced by 18.7% (*p* = 0.0159) and 27.6% (*p* = 0.0010); moreover, we found the GCS activity was higher than GPa activity in both HCC cells, the activity enhancement of GCS was 43.9% and 23.8% higher than that of GPa in HepG2 and HCCLM3 cells, respectively (Figure 2C). In contrast, compared to HepG2‐siNC and HCCLM3‐siNC, the GCS activity of HepG2‐siETV6 and HCCLM3‐siETV6 cells was weakened by 35.8% (*p* = 0.0003) and 34.9% (*p* = 0.0003), the GPa activity of HepG2‐siETV6 and HCCLM3‐siETV6 cells was weakened by 21.8% (*p* = 0.0022) and 14.9% (*p* = 0.0066); moreover, we found the GCS activity was lower than GPa activity in both HCC cells, the activity weakening of GCS was 13.9% and 20.0% lower than that of GPa in HepG2 and HCCLM3 cells, respectively (Figure 2C).

Furthermore, we examined the protein expression levels of glycogen metabolism‐related molecules. ETV6 overexpression increased the protein expression levels of p‐GSK3β by 40.7% (*p* = 0.0073) and 56.0% (*p* = 0.0005) in HepG2 and HCCLM3 cells, respectively (Figure 2D), while ETV6 overexpression decreased the protein expression levels of GSK3β by 36.3% (*p* < 0.0001) and 33.0% (*p* < 0.0001) in HepG2 and HCCLM3 cells, respectively (Figure 2D). Meanwhile, ETV6 silencing decreased the protein expression levels of p‐GSK3β by 40.7% (*p* = 0.0002) and 30.0% (*p* = 0.0028) in HepG2 and HCCLM3 cells, respectively (Figure 2D), while ETV6 silencing increased the protein expression levels of GSK3β by 28.7% (*p* = 0.0299) and 31.3% (*p* = 0.0038) in HepG2 and HCCLM3 cells, respectively (Figure 2D). In summary, these data robustly demonstrate that ETV6 facilitates glycogen synthesis in HCC cells.

### 3.3 miR‐429 Inhibits Glycolysis of HCC Cells

**FIGURE 3:**
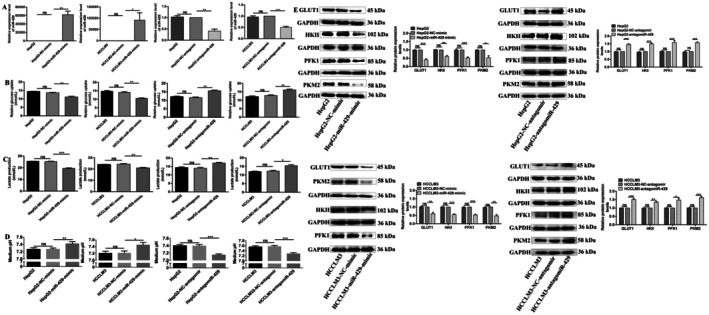
miR‐429 inhibits Warburg effect of HCC cells. (A) miR‐429 was overexpressed or downregulated in HepG2 and HCCLM3 cells. (B) Glucose uptake was measured in HepG2 and HCCLM3 cells by GOD‐POD assay. (C) Lactate production was measured in HepG2 and HCCLM3 cells by LOD assay. (D) The pH value in culture medium was measured in HepG2 and HCCLM3 cells. (E) The protein expression levels of glycolysis‐related molecules were measured in HepG2 and HCCLM3 cells by WB assay. *, **, *** Refer to *p* values < 0.05, 0.01, 0.001, ns refers to no statistical difference (*n* = 3).

To confirm the potential role of miR‐429 in the regulation of HCC cell glycolysis, we overexpressed and knocked down miR‐429 expression levels in HepG2 and HCCLM3 cells. miR‐429 expression levels were increased by 61,967‐fold (*p* = 0.0018) and 91,549‐fold (*p* = 0.0482) in HepG2‐miR‐429‐mimic and HCCLM3‐miR‐429‐mimic cells compared with HepG2‐NC‐mimic and HCCLM3‐NC‐mimic cells, respectively, while there were no expression level differences of miR‐429 between HepG2 and HepG2‐NC‐mimic or HCCLM3 and HCCLM3‐NC‐mimic cells (Figure 3A). Meanwhile, in comparison with HepG2‐NC‐antagomir and HCCLM3‐NC‐antagomir cells, miR‐429 expression levels decreased by 59.7% (*p* = 0.0024) and 50.3% (*p* = 0.0007) in HepG2‐antagomiR‐429 and HCCLM3‐antagomiR‐429 cells, respectively, while there were no expression level differences of miR‐429 between HepG2 and HepG2‐NC‐antagomir or HCCLM3 and HCCLM3‐NC‐antagomir cells (Figure 3A). The results above offer a controlled investigation into the impact of miR‐429 dysregulation on the glycolytic effect in HCC cells.

Firstly, GOD‐POD assay showed miR‐429 decreased glucose uptake of HCC cells. miR‐429 overexpression decreased the glucose uptake by 18.4% (*p* = 0.0097) and 24.8% (*p* = 0.0018) in both HepG2 and HCCLM3 cells (Figure 3B), in contrast, miR‐429 silencing increased the glucose uptake by 34.6% (*p* = 0.0014) and 26.0% (*p* = 0.0046) in both HepG2 and HCCLM3 cells (Figure 3B), while, there were no glucose consumption differences between HepG2 and HepG2‐NC‐mimic, HCCLM3 and HCCLM3‐NC‐mimic, HepG2 and HepG2‐NC‐antagomir or HCCLM3 and HCCLM3‐NC‐antagomir cells (Figure 3B).

Secondly, LOD assay showed miR‐429 decreased lactate production of HCC cells. The lactate production was decreased by 23.2% (*p* = 0.0003) and 14.0% (*p* = 0.0020) after miR‐429 overexpression in both HepG2 and HCCLM3 cells, while no lactate production differences were observed between HepG2 and HepG2‐NC‐mimic or HCCLM3 and HCCLM3‐NC‐mimic cells (Figure 3C). Meanwhile, miR‐429 silencing enhanced the lactate production by 21.6% (*p* = 0.0081) and 25.8% (*p* = 0.0131) in both HepG2 and HCCLM3 cells (Figure 3C), while there were no lactate production differences between HepG2 and HepG2‐NC‐antagomir or HCCLM3 and HCCLM3‐NC‐antagomir cells (Figure 3C). pH assay showed miR‐429 decreased culture medium acidity. In comparison with HepG2‐NC‐mimic and HCCLM3‐NC‐mimic, the pH value was increased in HepG2‐miR‐429‐mimic and HCCLM3‐miR‐429‐mimic cells culture medium (Figure 3D). While in comparison with HepG2‐NC‐antagomir and HCCLM3‐NC‐antagomir, the pH value was decreased in HepG2‐antagomiR‐429 and HCCLM3‐antagomiR‐429 cells culture medium (Figure 3D).

Furthermore, we examined the protein expression levels of glycolysis‐related molecules. miR‐429 overexpression decreased the protein expression levels of GLUT1, HKII, PFK1, PKM2 by 59.7% (*p* = 0.0007), 39.7% (*p* = 0.0072), 38.7% (*p* = 0.0018), and 45.0% (*p* = 0.0004), 48.0% (*p* = 0.0010) and 47.3% (*p* = 0.0005), 47.0% (*p* = 0.0174) and 52.7% (*p* = 0.0022) in HepG2 and HCCLM3 cells, respectively (Figure 3E). Meanwhile, miR‐429 silencing increased the protein expression levels of GLUT1, HKII, PFK1, PKM2 by 45.7% (*p* = 0.0008), 51.0% (*p* = 0.0009), 50.0% (*p* = 0.0002), and 34.3% (*p* = 0.0036), 58.7% (*p* = 0.0117) and 47.3% (*p* = 0.0162), 57.7% (*p* = 0.0003) and 62.0% (*p* = 0.0007) in HepG2 and HCCLM3 cells, respectively (Figure 3E). Collectively, these findings strongly demonstrate that miR‐429 suppresses the Warburg effect in hepatocellular carcinoma (HCC) cells.

### 3.4 miR‐429 Promotes Glycogen Synthesis of HCC Cells

**FIGURE 4:**
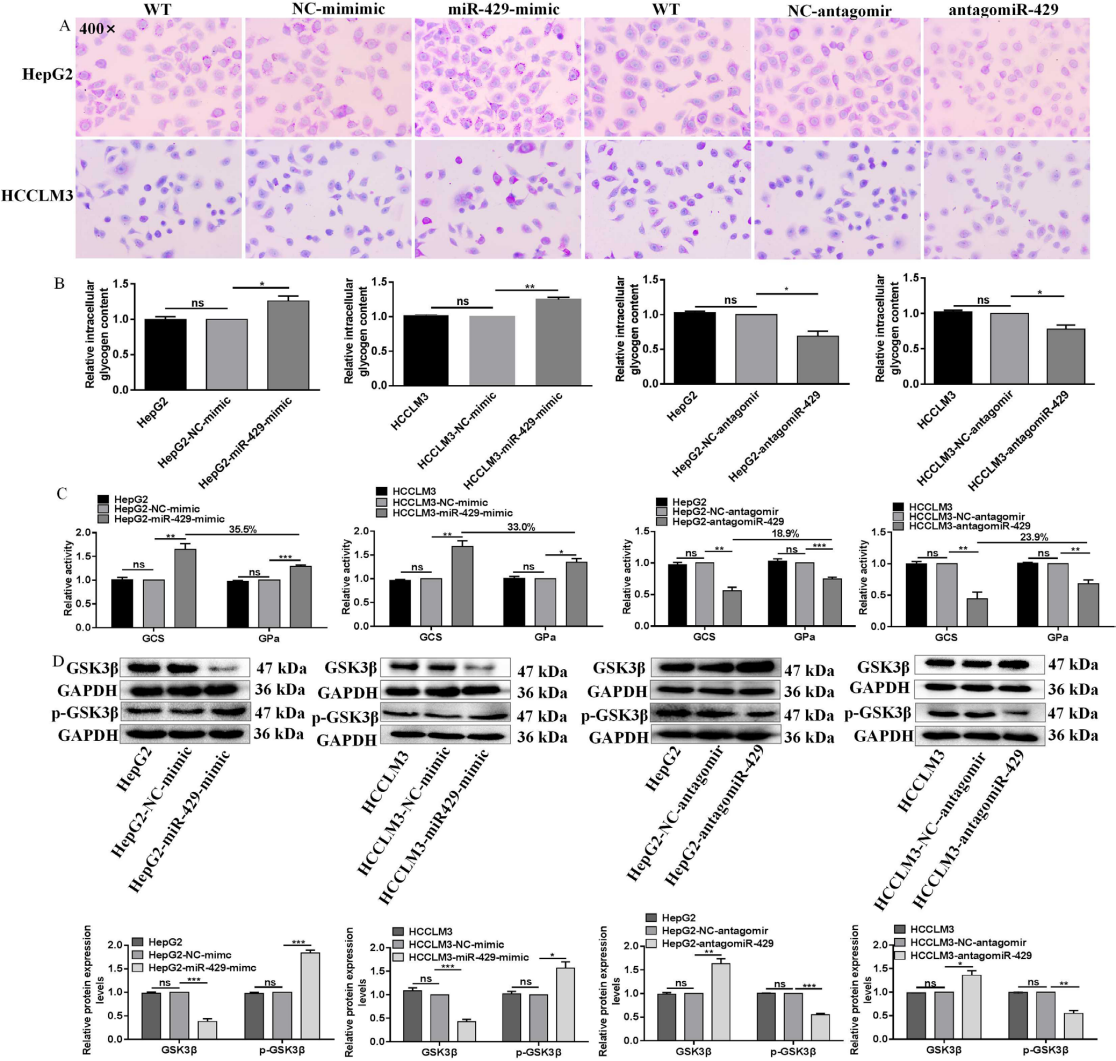
miR‐429 promotes glycogen synthesis of HCC cells. (A) Glycogen synthesis was measured in HepG2 and HCCLM3 cells by PAS assay. (B) Intracellular glycogen content was measured in HepG2 and HCCLM3 cells by anthrone assay. (C) The GCS and GPa activities were measured in HepG2 and HCCLM3 cells by NADH and NADPH assays. (D) The protein expression levels of glycogen metabolism‐related molecules were measured in HepG2 and HCCLM3 cells by WB assay. *, **, *** Refer to *p* values < 0.05, 0.01, 0.001, ns refers to no statistical difference (*n* = 3).

PAS assay showed miR‐429 promoted glycogen synthesis of HCC cells. Compared to HepG2‐NC‐mimic and HCCLM3‐NC‐mimic cells, HepG2‐miR‐429‐mimic and HCCLM3‐miR‐429‐mimic cells exhibited strong positive PAS reaction with amaranth spherical particle glycogen distributed in cytoplasm (Figure 4A). In contrast, compared to HepG2‐NC‐antagomir and HCCLM3‐NC‐antagomir, HepG2‐antagomiR‐429 and HCCLM3‐antagomiR‐429 cells exhibited weak positive PAS reaction with amaranth spherical particle glycogen reduced in cytoplasm (Figure 4A).

Anthrone assay showed miR‐429 increased the intracellular glycogen content of HCC cells. miR‐429 overexpression increased the intracellular glycogen content by 26.2% (*p* = 0.0195) and 24.7% (*p* = 0.0021) in both HepG2 and HCCLM3 cells (Figure 4B), in contrast, miR‐429 silencing decreased the intracellular glycogen content by 31.0% (*p* = 0.0118) and 22.1% (*p* = 0.0197) in both HepG2 and HCCLM3 cells (Figure 4B), while, there were no intracellular glycogen content differences between HepG2 and HepG2‐NC‐mimic, HCCLM3 and HCCLM3‐NC‐mimic, HepG2 and HepG2‐NC‐antagomir or HCCLM3 and HCCLM3‐NC‐antagomir cells (Figure 4B).

NADH and NADPH assays showed miR‐429 enhanced the GCS and GPa activities of HCC cells. Compared to HepG2‐NC‐mimic and HCCLM3‐NC‐mimic, the GCS activity of HepG2‐miR‐429‐mimic and HCCLM3‐miR‐429‐mimic cells was enhanced by 64.5% (*p* = 0.0074) and 67.7% (*p* = 0.0056), the GPa activity of HepG2‐miR‐429‐mimic and HCCLM3‐miR‐429‐mimic cells was enhanced by 29.0% (*p* = 0.0007) and 34.7% (*p* = 0.0122); moreover, we found the GCS activity was higher than GPa activity in both HCC cells, the activity enhancement of GCS was 35.5% (*p* = 0.0028) and 33.0% (*p* = 0.0367) higher than that of GPa, respectively (Figure 4C). In contrast, compared to HepG2‐NC‐antagomir and HCCLM3‐NC‐antagomir, the GCS activity of HepG2‐antagomiR‐429 and HCCLM3‐antagomiR‐429 cells was weakened by 42.2% (*p* = 0.0016) and 55.8% (*p* = 0.0066), the GPa activity of HepG2‐antagomiR‐429 and HCCLM3‐antagomiR‐429 cells was weakened by 25.3% (*p* = 0.0009) and 31.9% (*p* = 0.0070); moreover, we found the GCS activity was lower than GPa activity in both HCC cells, the activity weakening of GCS was 18.9% (*p* = 0.0208) and 23.9% (*p* = 0.0156) lower than that of GPa, respectively (Figure 4C).

Furthermore, we examined the protein expression levels of glycogen metabolism‐related molecules. miR‐429 overexpression increased the protein expression levels of p‐GSK3β by 83.7% (*p* = 0.0002) and 56.7% (*p* = 0.0130) in HepG2 and HCCLM3 cells, respectively (Figure 4D), while miR‐429 overexpression decreased the protein expression levels of GSK3β by 62.0% (*p* = 0.0005) and 57.0% (*p* = 0.0002) in HepG2 and HCCLM3 cells, respectively (Figure 4D). Meanwhile, miR‐429 silencing decreased the protein expression levels of p‐GSK3β by 45.0% (*p* = 0.0001) and 45.3% (*p* = 0.0021) in HepG2 and HCCLM3 cells, respectively (Figure 4D), while miR‐429 silencing increased the protein expression levels of GSK3β by 45.0% (*p* = 0.0001) and 45.3% (*p* = 0.0021) in HepG2 and HCCLM3 cells, respectively (Figure 4D). Taken together, the above results strongly support the notion that miR‐429 enhances the glycogen synthesis of HCC cells.

### 3.5 CRKL Promotes Glycolysis of HCC Cells

**FIGURE 5:**
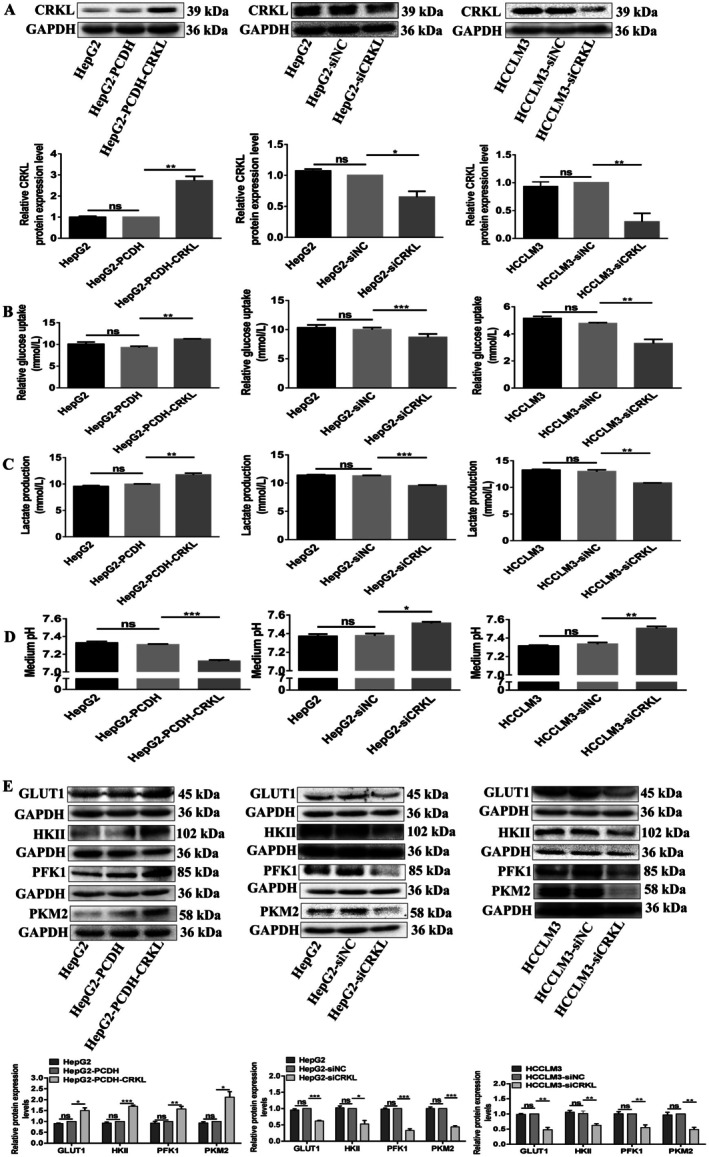
CRKL promotes Warburg effect of HCC cells. (A) CRKL were overexpressed or downregulated in HepG2 and HCCLM3 cells. (B) Glucose uptake was measured in HepG2 and HCCLM3 cells by GOD‐POD assay. (C) Lactate production was measured in HepG2 and HCCLM3 cells by LOD assay. (D) The pH value in culture medium was measured in HepG2 and HCCLM3 cells. (E) The protein expression levels of glycolysis‐related molecules were measured in HepG2 and HCCLM3 cells by WB assay. *, **, *** Refer to *p* values < 0.05, 0.01, 0.001, ns refers to no statistical difference (*n* = 3).

We previously investigated the effect of CRKL overexpression on glucose metabolism in HCCLM3 and Huh7 cells [33], in this article, we will delve into the effect of CRKL overexpression and silencing on glucose metabolism in HepG2 and HCCLM3 cells. CRKL protein level was increased by 172.7% (*p* = 0.0011) in HepG2‐PCDH‐ETV6 cells compared with HepG2‐PCDH cells, while there were no CRKL protein expression level differences between HepG2 and HepG2‐PCDH cells (Figure 5A). Meanwhile, in comparison with HepG2‐siNC and HCCLM3‐siNC cells, CRKL protein expression levels decreased by 38.3% (*p* = 0.0197) and 70.4% (*p* = 0.0013) in HepG2‐siCRKL and HCCLM3‐siCRKL cells, respectively, while there were no CRKL protein expression level differences between HepG2 and HepG2‐siNC or HCCLM3 and HCCLM3‐siNC cells (Figure 5A). The above results provide a control study for investigating CRKL deregulation on the glycolysis effect of HCC cells.

GOD‐POD assay showed CRKL enhanced glucose uptake of HCC cells. CRKL overexpression increased the glucose uptake by 20.9% (*p* = 0.0035) in HepG2 cells (Figure 5B), in contrast, CRKL silencing decreased the glucose uptake by 13.2% (*p* = 0.0008) and 31.0% (*p* = 0.0085) in both HepG2 and HCCLM3 cells (Figure 5B), while there were no glucose consumption differences between HepG2 and HepG2‐PCDH, HepG2 and HepG2‐siNC, or HCCLM3 and HCCLM3‐siNC cells (Figure 5B).

LOD assay showed CRKL enhanced lactate production of HCC cells. The lactate production was increased by 17.6% (*p* = 0.0062) after CRKL overexpression in HepG2 cells, while no lactate production difference was observed between HepG2 and HepG2‐PCDH cells (Figure 5C). Meanwhile, CRKL silencing decreased the lactate production by 15.4% (*p* = 0.0004) and 16.6% (*p* = 0.0026) in both HepG2 and HCCLM3 cells (Figure 5C), while there were no lactate production differences between HepG2 and HepG2‐siNC or HCCLM3 and HCCLM3‐siNC cells (Figure 5C). pH assay showed CRKL increased culture medium acidity. In comparison with HepG2‐PCDH, the pH value was decreased in HepG2‐PCDH‐ETV6 cells culture medium (Figure 5D). While in comparison with HepG2‐siNC and HCCLM3‐siNC, the pH value was increased in HepG2‐siETV6 and HCCLM3‐siETV6 cells culture medium (Figure 5D).

Furthermore, we examined the protein expression levels of glycolysis‐related molecules. CRKL overexpression increased the protein expression levels of GLUT1, HKII, PFK1, PKM2 by 49.7% (*p* = 0.0203), 69.3% (*p* = 0.0002), 57.0% (*p* = 0.0090), 112.0% (*p* = 0.0112) in HepG2 cells, respectively (Figure 5E). Meanwhile, CRKL silencing decreased the protein expression levels of GLUT1, HKII, PFK1, PKM2 by 41.0% (*p* = 0.0009) and 52.0% (*p* = 0.0028), 47.3% (*p* = 0.0119) and 37.3% (*p* = 0.0024), 67.3% (*p* = 0.0002) and 44.7% (*p* = 0.0069), 56.0% (*p* = 0.0001) and 51.3% (*p* = 0.0024) in HepG2 and HCCLM3 cells, respectively (Figure 5E). Taken together, the above results further strongly support the previous notion that CRKL enhances the Warburg effect of HCC cells.

### 3.6 CRKL Promotes Glycogen Synthesis of HCC Cells

**FIGURE 6:**
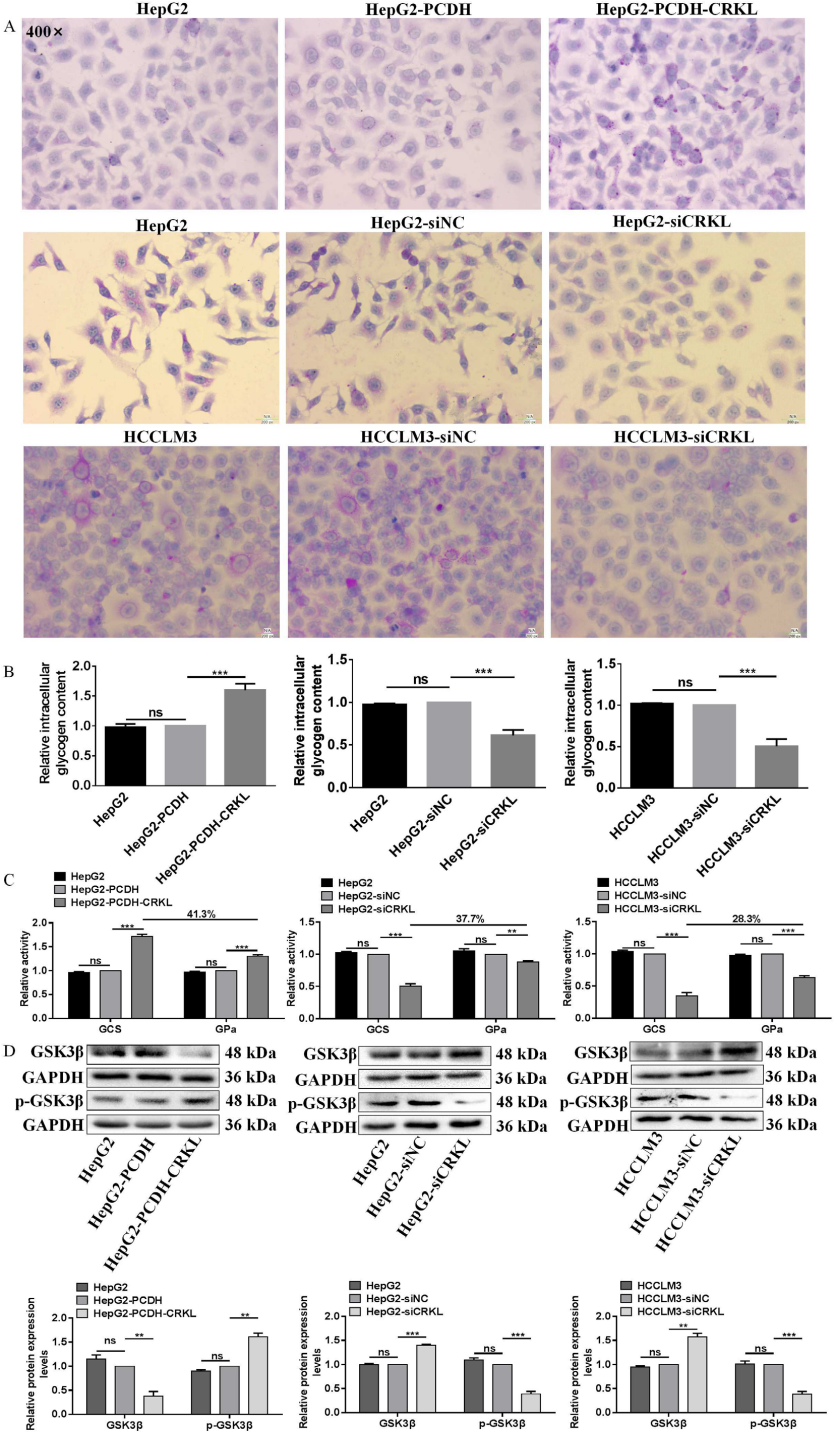
CRKL promotes glycogen synthesis of HCC cells. (A) Glycogen synthesis was measured in HepG2 and HCCLM3 cells by PAS assay. (B) Intracellular glycogen content was measured in HepG2 and HCCLM3 cells by anthrone assay. (C) The GCS and GPa activities were measured in HepG2 and HCCLM3 cells by NADH and NADPH assays. (D) The protein expression levels of glycogen metabolism‐related molecules were measured in HepG2 and HCCLM3 cells by WB assay. **, *** Refer to *p* values < 0.05, 0.01, 0.001, ns refers to no statistical difference (*n* = 3).

PAS assay showed CRKL promoted glycogen synthesis of HCC cells. Compared to HepG2‐PCDH, HepG2‐PCDH‐ETV6 cells exhibited a strong positive PAS reaction with amaranth spherical particle glycogen distributed in the cytoplasm (Figure 6A). In contrast, compared to HepG2‐siNC and HCCLM3‐siNC, HepG2‐siCRKL and HCCLM3‐siCRKL cells exhibited a weak positive PAS reaction with amaranth spherical particle glycogen reduced in the cytoplasm (Figure 6A).

Anthrone assay showed CRKL increased the intracellular glycogen content of HCC cells. CRKL overexpression increased the intracellular glycogen content by 59.9% (*p* = 0.0007) in HepG2 cells (Figure 6B), in contrast, CRKL silencing decreased the intracellular glycogen content by 38.5% (*p* = 0.0004) and 49.4% (*p* = 0.0005) in both HepG2 and HCCLM3 cells (Figure 6B), while there were no intracellular glycogen content differences between HepG2 and HepG2‐PCDH, HepG2 and HepG2‐siNC, or HCCLM3 and HCCLM3‐siNC cells (Figure 6B).

NADH and NADPH assays showed CRKL enhanced the GCS and GPa activities of HCC cells. Compared to HepG2‐PCDH, the GCS activity of HepG2‐PCDH‐CRKL cells was enhanced by 71.8% (*p* = 0.0001), the GPa activity of HepG2‐PCDH‐CRKL cells was enhanced by 30.3% (*p* = 0.0005); moreover, we found the GCS activity was higher than GPa activity, the activity enhancement of GCS was 41.3% higher than that of GPa, respectively (Figure 6C). In contrast, compared to HepG2‐siNC and HCCLM3‐siNC, the GCS activity of HepG2‐siCRKL and HCCLM3‐siCRKL cells was weakened by 49.3% (*p* = 0.0002) and 65.3% (*p* = 0.0002); the GPa activity of HepG2‐siCRKL and HCCLM3‐siCRKL cells was weakened by 11.78% (*p* = 0.0013) and 36.8% (*p* = 0.0002); moreover, we found the GCS activity was lower than GPa activity in both HCC cells, the activity weakening of GCS was 37.7% and 28.3% lower than that of GPa in HepG2 and HCCLM3 cells, respectively (Figure 6C).

Furthermore, we examined the protein expression levels of glycogen metabolism‐related molecules. CRKL overexpression increased the protein expression level of p‐GSK3β by 61.0% (*p* = 0.0012) in HepG2 cells (Figure 6D), while CRKL overexpression decreased the protein expression level of GSK3β by 62.0% (*p =* 0.0029) in HepG2 (Figure 6D). Meanwhile, CRKL silencing decreased the protein expression levels of p‐GSK3β by 61.0% (*p* = 0.0003) and 61.3% (*p* = 0.0004) in HepG2 and HCCLM3 cells, respectively (Figure 6D), while CRKL silencing increased the protein expression levels of GSK3β by 43.0% (*p* = 0.0003) and 57.3% (*p* = 0.0014) in HepG2 and HCCLM3 cells, respectively (Figure 6D). Taken together, the above results further strongly support the previous notion that CRKL enhances the glycogen synthesis of HCC cells.

### 3.7 ETV6 Binds to miR‐429 to Mediate Glucose Metabolic Reprogramming of HCC Cells by Targeting CRKL via PI3K/AKT Pathway

Previously, we discovered that the ETS domain of ETV6 directly binds to the GGAGGAAGCA sequence in the promoter region of miR‐429 (located at 696–705 bp site) to suppress its expression, miR‐429, in turn, inhibits CRKL expression by selectively targeting the CRKL‐3′‐UTR at the 3728–3735 bp site, meanwhile, ETV6 forms complexes with CRKL and promotes CRKL expression, thereby establishing an ETV6‐miR‐429‐CRKL regulatory loop [32], furthermore, CRKL regulates glucose metabolism in HCC cells via the PI3K/AKT pathway by directly binding to the p85 PxxP motif of PI3K [33]. We hypothesise that ETV6 binds to miR‐429 to mediate glucose metabolic reprogramming in HCC cells by targeting CRKL via PI3K/AKT pathway. Therefore, we investigated the effect of the ETV6‐miR‐429‐CRKL axis on PI3K/AKT pathway.

**FIGURE 7:**
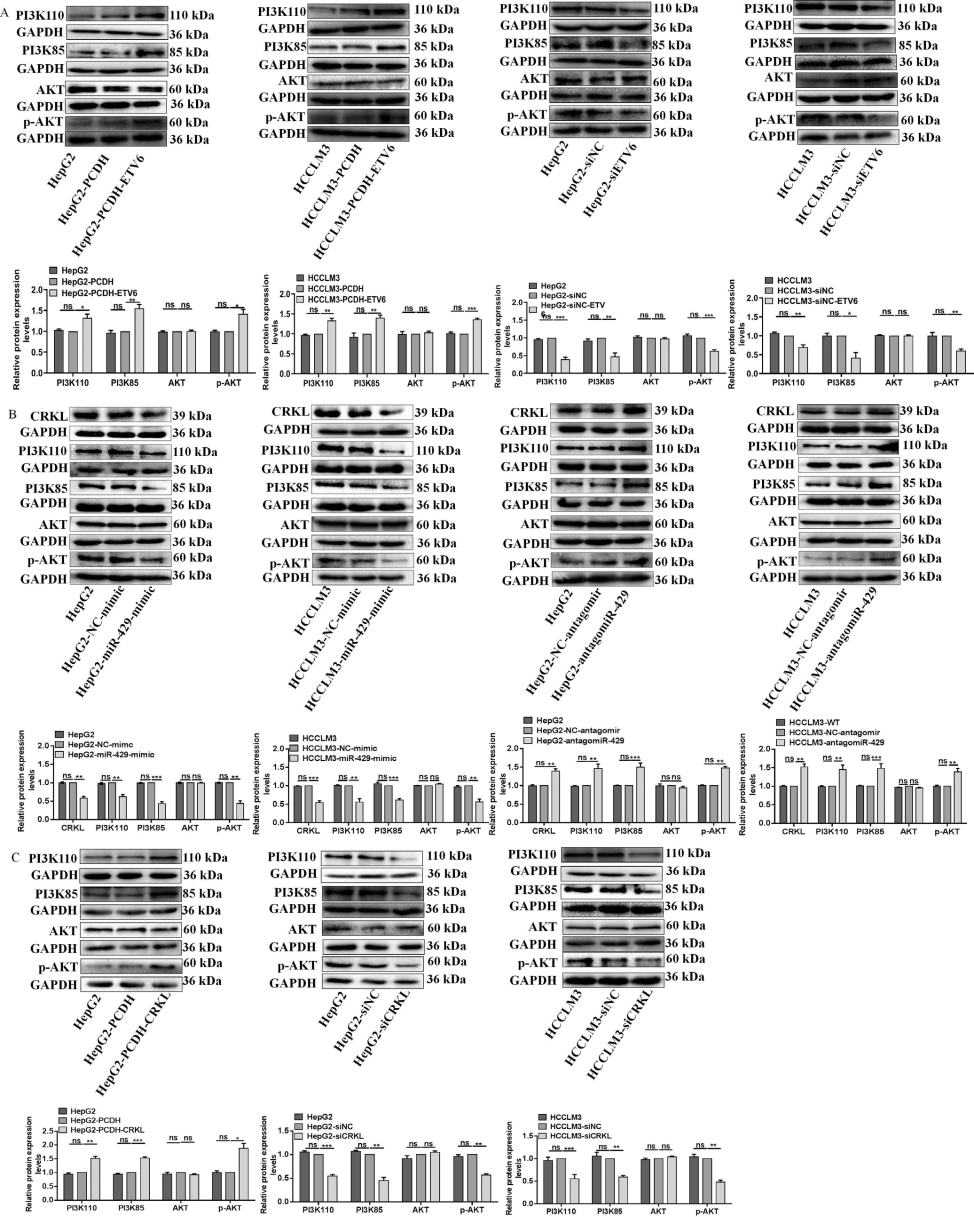
The ETV6‐miR‐429‐CRKL regulatory loop activated the PI3K/AKT pathway. (A) The influence of ETV6 deregulation on protein expression levels of PI3K110, PI3K85, p‐AKT, and AKT in HepG2 and HCCLM3 cells. (B) The influence of miR‐429 deregulation on protein expression levels of PI3K110, PI3K85, p‐AKT, and AKT in HepG2 and HCCLM3 cells. (C) The influence of CRKL deregulation on protein expression levels of PI3K110, PI3K85, p‐AKT, and AKT in HepG2 and HCCLM3 cells. *, **, *** Refer to *p* values < 0.05, 0.01, 0.001, ns refers to no statistical difference (*n* = 3).

ETV6 activated the PI3K/AKT pathway. ETV6 overexpression increased the protein expression levels of PI3K110, PI3K85, p‐AKT by 32.3% (*p* = 0.0234), 33.7% (*p* = 0.0033), 55.0% (*p* = 0.0047), and 40.7% (*p* = 0.0031), 42.3% (*p* = 0.0152), and 36.3% (*p* = 0.0002) in HepG2 and HCCLM3 cells, while there were no AKT protein expression level differences between HepG2 and HepG2‐PCDH or HCCLM3 and HCCLM3‐PCDH cells (Figure 7A). Conformably, ETV6 silencing decreased the protein expression levels of PI3K110, PI3K85, p‐AKT by 60.3% (*p* = 0.0005), 30.0% (*p* = 0.0087), 47.7% (*p* = 0.006), and 58.0% (*p* = 0.0139), 37.0% (*p* = 0.0003), and 39.3% (*p* = 0.0011) in HepG2 and HCCLM3 cells, while there were no AKT protein expression level differences between HepG2 and HepG2‐siNC or HCCLM3 and HCCLM3‐siNC cells (Figure 7A). The results indicate that ETV6 promotes glucose metabolism of HCC cells by activating the PI3K/AKT pathway.

miR‐429 inactivated the PI3K/AKT pathway. miR‐429 overexpression decreased the protein expression levels of PI3K110, PI3K85, p‐AKT by 37.7% (*p* = 0.0026), 44.3% (*p* = 0.0095), 56.3% (*p* = 0.0003) and 39.0% (*p* = 0.0006), 56.0% (*p* = 0.0013) and 44.0% (*p* = 0.0048) in HepG2 and HCCLM3 cells, while there were no AKT protein expression level differences between HepG2 and HepG2‐NC‐mimic or HCCLM3 and HCCLM3‐NC‐mimic cells (Figure 7B). Conformably, miR‐429 silencing increased the protein expression levels of PI3K110, PI3K85, p‐AKT by 46.3% (*p* = 0.0173), 44.7% (*p* = 0.0238), 49.7% (*p* = 0.0117) and 48.3% (*p* = 0.0194), 47.7% (*p* = 0.0004) and 38.7% (*p* = 0.0146) in HepG2 and HCCLM3 cells, while there were no AKT protein expression level differences between HepG2‐NC‐antagomir and HCCLM3‐NC‐antagomir cells (Figure 7B). The results indicate that miR‐429 inhibits glucose metabolism of HCC cells by inactivating the PI3K/AKT pathway.

We previously investigated the effect of CRKL overexpression on the PI3K/AKT pathway in HCCLM3 and Huh7 cells [33], in this article, we will delve into the effect of CRKL overexpression and silencing on the PI3K/AKT pathway in HepG2 and HCCLM3 cells. Consistent with previous results, CRKL overexpression increased the protein expression levels of PI3K110, PI3K85, p‐AKT by 51.3% (*p* = 0.0026), 53.0% (*p* = 0.0002), 88.3% (*p* = 0.0072) in HepG2 cells, while there were no AKT protein expression level differences between HepG2 and HepG2‐PCDH cells (Figure 7C). Conformably, CRKL silencing decreased the protein expression levels of PI3K110, PI3K85, p‐AKT by 45.3% (*p* = 0.0002) and 44.7% (*p* = 0.0097), 54.7% (*p* = 0.0012) and 41.0% (*p* = 0.0003), 38.7% (*p* = 0.0033) and 52.0% (*p* = 0.0002) in HepG2 and HCCLM3 cells, while there were no AKT protein expression level differences between HepG2 and HepG2‐siNC or HCCLM3 and HCCLM3‐siNC cells (Figure 7C). The results further prove that CRKL promotes glucose metabolism of HCC cells by activating the PI3K/AKT pathway. According to the above results and our previous findings, it is clear that ETV6 binds to miR‐429 to mediate glucose metabolic reprogramming of HCC cells by targeting CRKL via the PI3K/AKT pathway.

### 3.8 ETV6‐miR‐429‐CRKL Regulatory Loop Promotes in Vivo Glucose Metabolism of HCC Cells

Previously, we successfully constructed the nude mice xenograft model with stable ETV6, CRKL knockdown and miR‐429 upregulation. We found ETV6, CRKL and miR‐429 affect the in vitro glucose metabolism of HCC cells, and whether the three molecules affect the in vivo glucose metabolism of HCC cells; thus, we further investigate the effect of ETV6, CRKL and miR‐429 on in vivo glucose metabolism of HCC cells.

**FIGURE 8:**
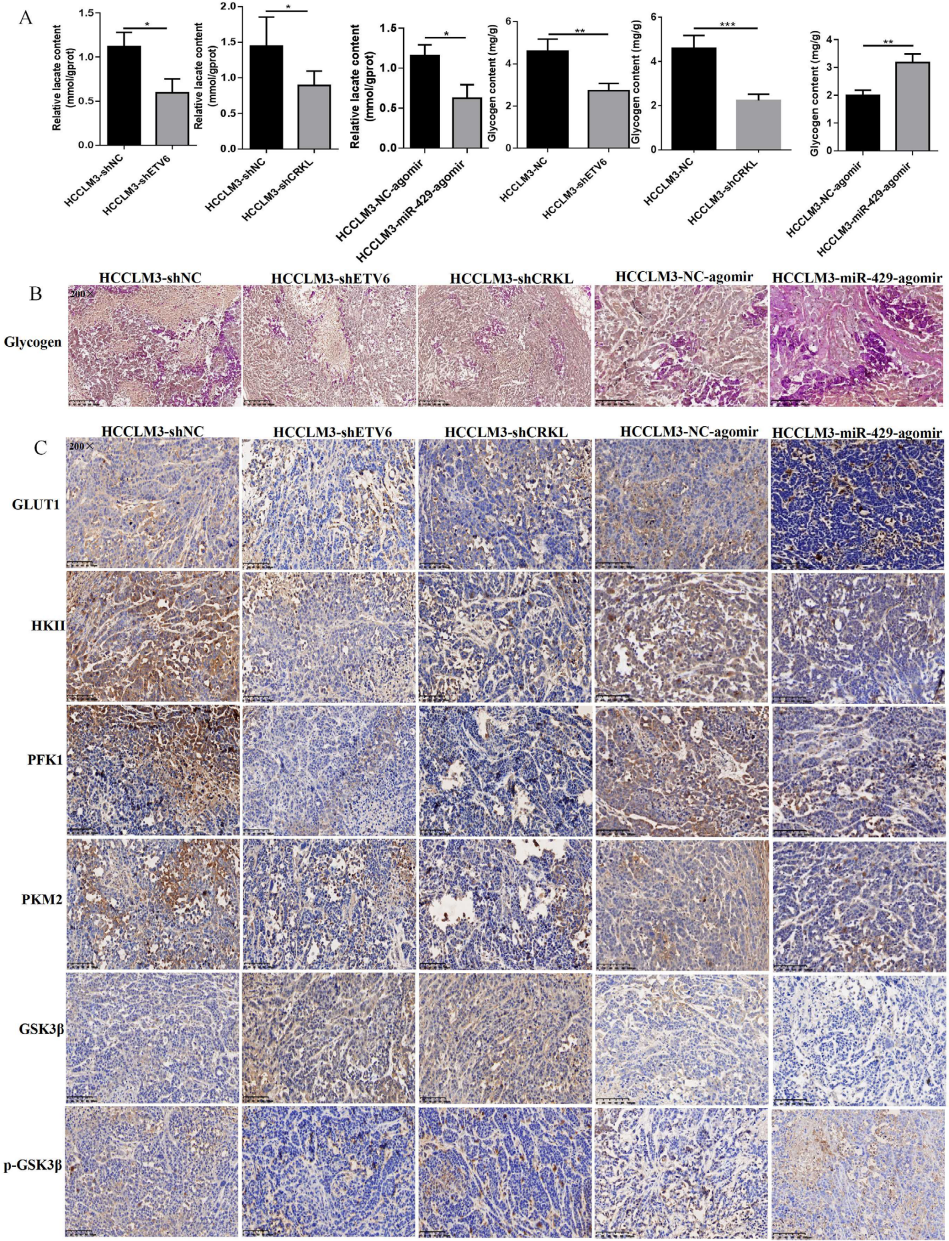
ETV6, CRKL and miR‐429 affect the in vivo glucose metabolism of HCC cells. (A) The effect of ETV6, CRKL knockdown and miR‐429 overexpression on lactate production and glycogen content of HCCLM3‐transplanted mice by LOD and anthrone assays. (B) The effect of ETV6, CRKL knockdown and miR‐429 overexpression on glycogen synthesis of HCCLM3‐transplanted mice by PAS assay. (C) The effect of ETV6, CRKL knockdown and miR‐429 overexpression on glucose metabolism‐related molecules expression level by IHC assay. *, **, ***Refer to *p* values < 0.05, 0.01, 0.001, ns refers to no statistical difference (*n* = 5).

In consistent with the in vitro results, ETV6 and CRKL knockdown or miR‐429 overexpression decreased lactate production of HCCLM3 cells in vivo. The lactate content was decreased by 52.6% (*p* = 0.0159), 55.6% (*p* = 0.0012) and 52.9% (*p* = 0.0131) in HCCLM3‐shETV6‐transplanted, HCCLM3‐shCRKL‐transplanted and HCCLM3‐miR‐429‐agomir‐transplanted mice compared with HCCLM3‐shNC‐transplanted and HCCLM3‐NC‐agomir‐transplanted mice (Figure 8A).

Meanwhile, ETV6 and CRKL knockdown inhibited glycogen synthesis and miR‐429 overexpression promoted glycogen synthesis of HCCLM3 cells in vivo. The glycogen content was decreased by 40.5% (*p* = 0.0014) and 51.2% (*p* = 0.0003) in HCCLM3‐shETV6‐transplanted and HCCLM3‐shCRKL‐transplanted mice compared with HCCLM3‐shNC‐transplanted mice; moreover, the glycogen content was increased by 58.5% (*p* = 0.0047) in HCCLM3‐miR‐429‐agomir‐transplanted mice compared with HCCLM3‐NC‐agomir‐transplanted mice (Figure 8A). Moreover, HCCLM3‐shETV6‐transplanted and HCCLM3‐shCRKL‐transplanted mice exhibited weak positive PAS reaction, with amaranth spherical particle glycogen reduced in cytoplasm compared with HCCLM3‐shNC‐transplanted mice, while HCCLM3‐miR‐429‐agomir‐transplanted mice exhibited strong positive PAS reaction, with amaranth spherical particle glycogen distributed in cytoplasm compared with HCCLM3‐NC‐agomir‐transplanted mice (Figure 8B).

Furthermore, IHC assays were further performed to measure the protein expression levels of glycolysis‐ and glycogen metabolism‐related molecules in mice tumour tissues. The expression levels of GLUT1, HKII, PFK1 and PKM2 in primary tumour from HCCLM3‐shETV6‐transplanted, HCCLM3‐shCRKL‐transplanted and HCCLM3‐miR‐429‐agomir‐transplanted mice were lower than that in the HCCLM3‐shNC‐transplanted and HCCLM3‐NC‐agomir‐transplanted mice, while, The expression levels of GSK3β and p‐GSK3β were increased and decreased in HCCLM3‐shETV6‐transplanted or HCCLM3‐shCRKL‐transplanted mice, respectively, the expression levels of GSK3β and p‐GSK3β were decreased and increased in HCCLM3‐miR‐429‐agomir‐transplanted mice (Figure 8C). The results further demonstrate ETV6 and CRKL knockdown inhibit glycolysis and glycogen synthesis of HCCLM3 cells in vivo, miR‐429 overexpression inhibits glycolysis and promotes glycogen synthesis of HCCLM3 cells in vivo. The effect of ETV6, CRKL and miR‐429 on the in vivo tumorigenicity of HCC cells via affecting glucose metabolism.

**FIGURE 9:**
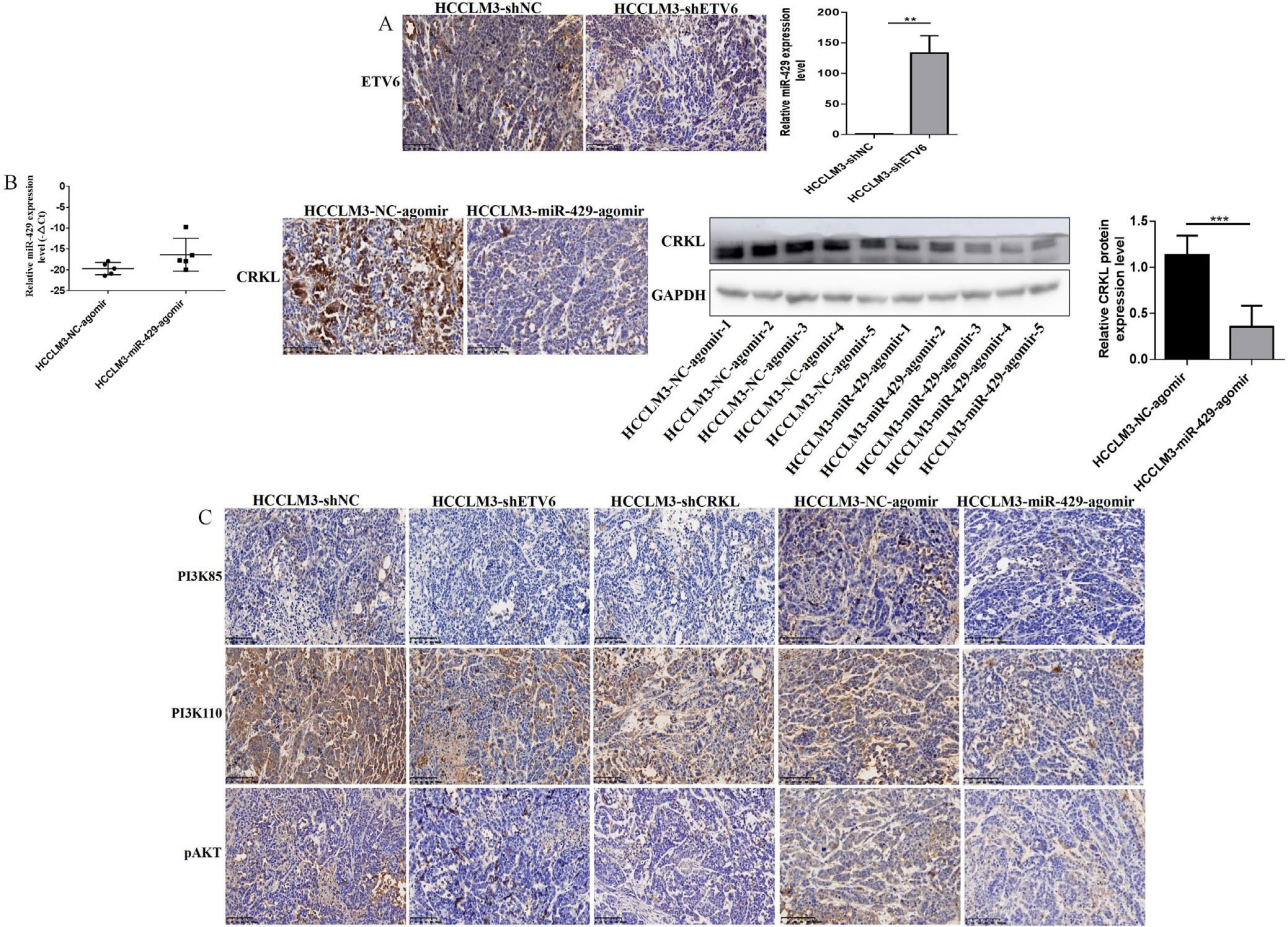
ETV6‐miR‐429‐CRKL regulatory loop mediates in vivo glucose metabolism of HCC cells via the PI3K/AKT pathway. (A) ETV6 downregulation increases the miR‐429 expression level in HCCLM3‐shETV6‐transplanted mice. (B) miR‐429 overexpression decreases the CRKL expression level in HCCLM3‐miR‐429‐agomir‐transplanted mice. (C) The influence of ETV6/CRKL knockdown and miR‐429 overexpression on protein expression levels of PI3K110, PI3K85 and p‐AKT in vivo. **, ***Refer to *p* values < 0.01, 0.001, ns refers to no statistical difference (*n* = 5).

ETV6‐miR‐429‐CRKL regulatory loop mediates in vivo glucose metabolism of HCC cells via PI3K/AKT pathway. Previously, we found ETV6 negatively regulates miR‐429 expression by directly binding to its promoter region in vitro [32], we also found the negative correlation between ETV6 and miR‐429 in vivo, compared with HCCLM3‐shNC‐transplanted mice, the expression level of miR‐429 was increased by 133.4‐fold (*p* = 0.0013) in HCCLM3‐shETV6‐transplanted mice (Figure 9A). Our results further demonstrate that ETV6 expression was negatively correlated with miR‐429 expression. Moreover, miR‐429 negatively regulates CRKL expression by selectively targeting CRKL‐3′‐UTR [32], we also found the negative correlation between miR‐429 and CRKL in vivo, the expression level of CRKL in primary tumour from HCCLM3‐miR‐429‐agomir‐transplanted mice was lower than that in the HCCLM3‐NC‐agomir‐transplanted mice by IHC assay, meanwhile, compared with HCCLM3‐NC‐agomir‐transplanted mice, the expression level of CRKL was decreased by 68.7% (*p* = 0.0005) in HCCLM3‐miR‐429‐agomir‐transplanted mice by WB assay (Figure 9B). Our results further demonstrate that miR‐429 expression was negatively correlated with CRKL expression.

Since ETV6, CRKL, miR‐429 affect the in vitro glucose metabolism of HCC cells by regulating the PI3K/AKT pathway, we further investigate the impact of ETV6, CRKL, miR‐429 dysregulation on the PI3K/AKT pathway in vivo. Consistent with the in vitro findings, ETV6/CRKL knockdown and miR‐429 overexpression decreased the protein expression levels of PI3K110, PI3K85, p‐AKT in HCCLM3‐shETV6‐transplanted, HCCLM3‐shCRKL‐transplanted and HCCLM3‐miR‐429‐agomir‐transplanted mice compared to HCCLM3‐shNC‐transplanted and HCCLM3‐NC‐agomir‐transplanted mice (Figure 9C). These results demonstrate that ETV6 binds to miR‐429 to mediate in vivo glucose metabolic reprogramming in HCC cells by targeting CRKL via the PI3K/AKT pathway.

## 4 Discussion

Reprogramming of cancer metabolism is a newly recognised hallmark of malignancy. The aberrant glucose metabolism is associated with dramatically increased bioenergetic, biosynthetic, and redox demands, which are vital to maintain rapid cell proliferation, tumour progression, meet biosynthetic needs, adapt to various microenvironments and resistance to chemotherapy and radiation. Aberrant glucose metabolism could alter many physiological activities, inducing DNA damage repair, enhancing autophagy, changing the tumour microenvironment and increasing the secretion of exosomes [34]. Cancer cells characterised by uncontrolled growth and proliferation require altered metabolic processes to maintain this characteristic. Metabolic reprogramming is a process mediated by various factors, including oncogenes, tumour suppressor genes, changes in growth factors, and tumour‐host cell interactions, which help to meet the needs of cancer cell anabolism and promote tumour development [35, 36]. Metabolic reprogramming in tumour cells is dynamically variable, depending on the tumour type and microenvironment, and reprogramming involves multiple metabolic pathways. These metabolic pathways have complex mechanisms and involve the coordination of various signalling molecules, proteins, and enzymes, which increase the resistance of tumour cells to traditional antitumor therapies. With the development of cancer therapies, metabolic reprogramming has been recognised as a new therapeutic target for metabolic changes in tumour cells. Therefore, a better understanding of the molecular mechanism underlying glucose metabolism is urgently needed in order to discover novel therapeutic targets and strategies to fight tumours [37].

ETV6 and CRKL deregulation is associated with various cancers, which is an interesting biomarker for diagnosis, therapy and prognosis of tumours. Previously, we found that ETV6 and CRKL potentially promoted hepatocarcinogenesis and metastasis of HCC cells, we also found that CRKL promoted glucose metabolism of HCC cells, meanwhile, ETV6 directly binds to CRKL and positively its expression [32, 33]. Furthermore, dysregulated glucose metabolism of tumour cell provides an acidic microenvironment that facilitates tumour cell metastasis, based on these findings, we speculate that ETV6 may affect the glucose metabolism of HCC cells. So, in the current study, we investigated the potential role and underlying mechanism of ETV6 in glucose metabolism of HCC in vitro and in vivo.

In the 1920s, Otto Warburg first showed that, unlike normal cells, which catabolise glucose by mitochondria oxidative phosphorylation, cancer cells tend to convert glucose into lactate even in the presence of sufficient oxygen [38, 39]. This phenomenon was termed as aerobic glycolysis or the Warburg effect, and is characterised by enhanced glucose uptake and lactate production. Previously, we only investigated the effect of CRKL overexpression on the glucose metabolism of HCCLM3 and Huh7 cells, so, in this article, we will further measure the effect of CRKL overexpression and knockdown on the glucose metabolism of HCCLM3 and HepG2 cells. We found that ETV6 and CRKL overexpression enhanced the glucose uptake of HepG2 and HCCLM3 cells; in contrast, ETV6 and CRKL knockdown inhibited the glucose uptake of HepG2 and HCCLM3 cells (Figures 1B, 5B). Meanwhile, ETV6 and CRKL overexpression enhanced lactate production of HepG2 and HCCLM3 cells, while ETV6 and CRKL knockdown inhibited lactate production of HepG2 and HCCLM3 cells (Figures 1C, 5C). The lactate production provides an acidic environment to aid the invasion and metastasis of the tumour, resulting in a decreased pH value of the culture medium. ETV6 and CRKL overexpression decreased the pH value of HepG2 and HCCLM3 cell culture medium; conformably, ETV6 and CRKL knockdown increased the pH value of HepG2 and HCCLM3 cell culture medium (Figures 1D, 5D). Glucose is not able to cross the plasma membrane on its own due to the hydrophilic composition of glucose; therefore, in order to overcome this condition, cancer cells induce GLUT1 expression. GLUT1 is a transmembrane protein for regulating the entry of extracellular glucose across the plasma membrane into cells during the glucose metabolism process. GLUT1 has a high affinity for glucose and is overexpressed in various tumours [40]. We found ETV6 and CRKL overexpression increased the protein expression level of GLUT1 in HepG2 and HCCLM3 cells; ETV6 and CRKL knockdown decreased the protein expression level of GLUT1 in HepG2 and HCCLM3 cells (Figures 1E, 5E).

Glucose metabolic enzymes and metabolites regulate gene expression through multiple mechanisms: (1) their intrinsic protein kinase activities; (2) direct interactions with transcription factors; (3) modulation of transcriptional co‐regulators (e.g., activators, repressors, and histone modifiers); and (4) control of mRNA stability, splicing, and translation. These processes play a critical role in both normal physiology and tumour development [41, 42]. There are three rate‐limiting enzymes in aerobic glycolysis, including HK, PFK, and PKs; the expression changes of the three enzymes can widely influence the development and progression of tumour. HK is the first rate‐limiting enzyme in aerobic glycolysis and can catalyse glucose transformed into G‐6‐P. There are four isoforms of HK (HKI, HKII, HKIII, and HKIV); HKII is upregulated in various tumours and it is directly linked to the pathological stage and patient prognosis of tumour [43]. HKII is closely involved in glycolysis; it can promote glucose metabolism to meet the energy needs of cancer cells, inhibit the accumulation of ROS in mitochondria, and increase the adaptability and survival of cancer cells [44]. The second committed step of glycolysis is catalysed by PFK1, which catalyses the conversion of fructose‐6 phosphate (F‐6‐P) into fructose‐1,6‐bisphosphate (F‐1,6‐2P); increased PFK1 activity can promote glycolysis and proliferation of tumour cells [45]. The third committed step is catalysed by PKM2, which catalyses phosphoenolpyruvate (PEP) to pyruvate; it plays a crucial role in promoting the progression of cancer [46]. We found that ETV6 and CRKL overexpression increased the protein expression level of HKII, PFK1, and PKM2 in HepG2 and HCCLM3 cells; while ETV6 and CRKL knockdown decreased the protein expression level of HKII, PFK1, and PKM2 in HepG2 and HCCLM3 cells (Figures 1E, 5E). Taken together, our findings strongly support the notion that ETV6 and CRKL enhance the Warburg effect of HCC cells.

Liver is a vital organ involved in glycogen synthesis, glucose metabolism and blood glucose maintenance. Glycogen, a glucose polymer, serves as a storage form of glucose; its structure consists of long polymetric chains of glucose units linked by α‐1,4 glycosidic bonds, with occasional α‐1,6 glycosidic bonds that generate branching points and increase solubility. Glycogen metabolism plays a crucial role in maintaining glucose and energy metabolism homeostasis [47]. When cancer cells encounter transient energy deficits, they utilise glycogen stores to supply glucose for glycolysis. ETV6 and CRKL overexpression promoted glycogen synthesis and increased glycogen content of HepG2 and HCCLM3 cells, while ETV6 and CRKL knockdown inhibited glycogen synthesis and decreased glycogen content of HepG2 and HCCLM3 cells (Figures 2A,B, 6A,B). The synthesis and utilisation of glycogen require the coordinated action of a series of enzymes. The deficiency of any of these enzymes could induce glycogen synthesis and utilisation dysfunctions. GCS and GPa catalyse glycogen synthesis and degradation, respectively [48]. GCS elongates the glucose chain by attaching uridine diphosphate (UDP)‐glucose units through α‐1,4 glycocidic linkage; GPa is the rate‐limiting enzyme of glycogenolysis, cleaving the α‐1,4 linkage to remove glucose residues from the glycogen chain as glucose‐1‐phosphate [49]. GSK3β is the primary rate‐limiting enzyme for glycogen synthesis through phosphorylating and inactivating GCS; GSK3β can also autophosphorylate, leading to the inactivation of the kinase. The inactivated GSK3β is unable to phosphorylate and inactivate GCS; subsequently, glycogen is synthesised by GCS [50, 51]. GSK3β plays an important role in the mediation of blood glucose homeostasis. We found that ETV6 and CRKL overexpression increased/decreased the protein expression level of GSK3β/p‐GSK3β in HepG2 and HCCLM3 cells; meanwhile, ETV6 and CRKL knockdown decreased/increased the protein expression level of GSK3β/p‐GSK3β in HepG2 and HCCLM3 cells (Figures 2D, 6D). Meanwhile, ETV6 and CRKL overexpression increased the GCS and GPa activities of HepG2 and HCCLM3 cells; ETV6 and CRKL knockdown decreased the GCS and GPa activities of HepG2 and HCCLM3 cells, while the degree of increase and decrease of GCS activity was larger than GPa activity in both HCC cells (Figures 2C, 6C). Our findings strongly support the notion that ETV6 and CRKL enhance the glycogen synthesis of HCC cells.

Previously, we demonstrated that the ETS domain of ETV6 directly binds to the GGAGGAAGCA sequence in the miR‐429 promoter region (696–705 bp site) to suppress its expression [32], in this study, ETV6 could regulate the glucose metabolism of HCC cells, so, we speculate miR‐429 maybe also affect the glucose metabolism of HCC cells. Our results showed that miR‐429 overexpression decreased the glucose uptake and lactate production of HepG2 and HCCLM3 cells, in contrast, miR‐429 knockdown increased the glucose uptake and lactate production of HepG2 and HCCLM3 cells (Figure 3B,C). Conformably, miR‐429 overexpression increased the pH value of HepG2 and HCCLM3 cells, miR‐429 knockdown decreased the pH value of HepG2 and HCCLM3 cells (Figure 3D). Furthermore, miR‐429 overexpression decreased the protein expression level of HKII, PFK1 and PKM2 in HepG2 and HCCLM3 cells, while, miR‐429 knockdown increased the protein expression level of HKII, PFK1 and PKM2 in HepG2 and HCCLM3 cells (Figure 3E). Taken together, our findings strongly support the notion that miR‐429 inhibits the Warburg effect of HCC cells.

We further investigate the effect of miR‐429 deregulation on glycogen synthesis. Interestingly, miR‐429 overexpression promoted glycogen synthesis and increased glycogen content of HepG2 and HCCLM3 cells; miR‐429 knockdown inhibited glycogen synthesis and decreased glycogen content of HepG2 and HCCLM3 cells (Figure 4A,B). Meanwhile, we found that miR‐429 overexpression increased/decreased the protein expression level of GSK3β/p‐GSK3β in HepG2 and HCCLM3 cells; meanwhile, miR‐429 knockdown decreased/increased the protein expression level of GSK3β/p‐GSK3β in HepG2 and HCCLM3 cells (Figure 4D). Meanwhile, miR‐429 overexpression increased the GCS and GPa activities of HepG2 and HCCLM3 cells; miR‐429 knockdown decreased the GCS and GPa activities of HepG2 and HCCLM3 cells, while the degree of increase and decrease of GCS activity was higher than GPa activity in both HCC cells (Figure 4C). Our findings indicate miR‐429 also enhances the glycogen synthesis of HCC cells; this is not consistent with the negative regulatory correlation of ETV6 with miR‐429; we believe that this phenomenon is related to glycogen shunt. Glycogen metabolism presents significant biological roles through diverse metabolic pathways beyond its effect as a reservoir of glucose, including glycolysis and PPP pathways. The glycogen shunt is described as a condition when glucose is shunted to glycogen and subsequently consumed through glycolysis even under the condition of adequate glucose, thereby coupling glycogen synthesis and breakdown pathways to glycolysis, which plays a vital role in tumorigenesis, development, and cell survival of tumours [52]. The coordinated action of the glycogen shunt and glycolysis allows the cells to store glucose as glycogen while maintaining homeostasis of glycolytic intermediates and ATP and supplying glycolytic substrates for the PPP when needed. The storage of excess glucose intake as glycogen allows energy to be preserved for future use, even though efficiency is slightly reduced by the net production of lactate [53]. In the current study, miR‐429 can mediate glycogen shunt by influencing the activities of GCS and GPa; miR‐429 overexpression enhanced the activities of GCS and GPa; the GCS activity was higher than GPa activity in both HCC cells, thereby promoting glycogen synthesis; meanwhile, miR‐429 knockdown weakened the activities of GCS and GPa; the GPa activity was higher than GCS activity in both HCC cells, thereby promoting glycogen synthesis; glycogen tends to degradation into the aerobic glycolysis pathway, which is enhanced to provide more nutrients and energy for tumour cells and promote the progression of HCC. Aerobic glycolysis and glycogen shunt processes are coordinated to achieve more efficient use of glucose and maintain the metabolic balance of cancer cells. Based on the importance of the glycogen shunt, supporting flow into and out of the glycogen pool, modifying GCS or GPa would make cancer cells vulnerable to free radical damage by blocking G‐6‐P production from glycogen [54].

The PI3K/AKT signalling axis serves as a central regulator of glucose metabolism through multiple mechanisms: (1) direct phosphorylation of key metabolic effectors including GLUT1, HK2, PFKB3/4 and PKM2; (2) coordinated control of critical regulatory nodes such as mTORC1, GSK3, FOXO family transcription factors, MYC and HIF‐1α. This comprehensive metabolic reprogramming extends beyond glycolysis to modulate the pentose phosphate pathway, mitochondrial oxidative phosphorylation, lipogenesis and redox balance, thereby simultaneously satisfying the bioenergetic and biosynthetic requirements of proliferating tumour cells [10, 55]. AKT facilitates the translocation of GLUT1 to the plasma membrane, enhancing cellular glucose uptake. Additionally, AKT targets HK2, mediating its binding to the voltage‐dependent anion channel (VDAC) on the outer mitochondrial membrane; this interaction enables HK2 to utilise mitochondrially derived ATP directly, thereby activating glucose‐6‐phosphate production. Meanwhile, the PI3K/AKT cascade enhances nucleotide synthesis by inducing Glucose‐6‐phosphate dehydrogenase (G6PD) activity, while it can further enhance glycolysis by targeting 6‐phosphofructo‐2‐kinase/fructose‐2,6‐bisphosphatase (PFKFB), which generates fructose‐2,6‐bisphosphate to activate PFK1 and AKT directly interacts with PKM2 by phosphorylating it at Ser202 [10, 56]. Beyond glycolysis, AKT promotes glycogen synthesis by phosphorylating and inactivating GSK3β [57]. The activation of PI3K is initiated when the SH3N domain of CRKL binds to the p85 PxxP motif of PI3K, triggering the production of phosphoinositides PIP2 and PIP3 [58]. Subsequently, AKT binds to PIP3 via its pleckstrin homology (PH) domain and undergoes phosphorylation at Thr308 by phosphoinositide‐dependent kinase‐1 (PDK1), which also binds PIP3. This activation enables AKT to phosphorylate downstream substrates, eliciting diverse biological effects [59]. Our results showed that ETV6 and CRKL overexpression increased the protein expression levels of PI3K110, PI3K85, p‐AKT in HepG2 and HCCLM3 cells; ETV6 and CRKL knockdown decreased the protein expression levels of PI3K110, PI3K85, p‐AKT in HepG2 and HCCLM3 cells (Figure 7A,C), miR‐429 overexpression decreased the protein expression levels of PI3K110, PI3K85, p‐AKT in HepG2 and HCCLM3 cells; miR‐429 silencing increased the protein expression levels of PI3K110, PI3K85, p‐AKT in HepG2 and HCCLM3 cells (Figure 7B). Based on the above results and our previous findings regarding the correlation among ETV6, miR‐429 and CRKL, it can be concluded that ETV6 binds to miR‐429 to mediate glucose metabolic reprogramming in HCC cells by targeting CRKL via the PI3K/AKT pathway. Emerging evidence highlights that hyperactivation of the PI3K/Akt pathway promotes tumorigenesis and disease progression by reprogramming glucose metabolism. Future research should prioritise: systematic mapping of key regulatory nodes linking PI3K/Akt signalling to glycolytic flux across cancer types, uncovering novel metabolic vulnerabilities; elucidating crosstalk between PI3K/Akt and other glycolysis‐associated pathways to fully understand the oncogenic consequences of sustained Akt activation. Given their druggable nature and critical role in sustaining cancer cell proliferation, metabolic enzymes represent promising therapeutic targets. Deciphering these mechanisms may reveal innovative strategies to disrupt tumour metabolic dependencies. Notably, the tumour microenvironment—including stromal heterogeneity in primary and metastatic lesions and host nutritional status—modulates the metabolic effects of PI3K/Akt signalling. These context‐dependent interactions reinforce the rationale for combining metabolic therapies in cancer treatment.

We have confirmed the effect of the ETV6‐miR‐429‐CRKL regulatory loop on glucose metabolism of HCC cells in vitro, we will further verified its potential role on tumorigenic ability and glucose metabolism of HCCLM3 cells in vivo. We successfully constructed the nude mice xenograft model with ETV6, CRKL knockdown and miR‐429 overexpression, ETV6 and CRKL knockdown or miR‐429 overexpression decreased the in vivo tumorigenicity of HCCLM3 cells, to prevent duplicate publication, we have excluded the tumour growth curves and representative tumour images from this manuscript, as these data are part of a separate study currently under review at the International Journal of Oncology. Consistent with the in vitro results, ETV6 and CRKL knockdown or miR‐429 overexpression decreased lactate production of HCCLM3 cells in vivo (Figure 8A), Meanwhile, ETV6 and CRKL knockdown inhibited glycogen synthesis and miR‐429 overexpression promoted glycogen synthesis of HCCLM3 cells in vivo (Figure 8A,B). Furthermore, IHC assays showed that ETV6 and CRKL knockdown or miR‐429 overexpression decreased the expression levels of glycolysis‐related molecules of GLUT1, HKII, PFK1 and PKM2 in tumour tissues from HCCLM3‐transplanted mice, while, ETV6/CRKL knockdown increased and decreased the expression levels of glycogen metabolism‐related molecules of GSK3β and p‐GSK3β in tumour tissues from HCCLM3‐transplanted mice, miR‐429 overexpression decreased and increased the expression levels of GSK3β and p‐GSK3β in tumour tissues from HCCLM3‐transplanted mice (Figure 8C). Previously, we have found the regulatory relationship among the three molecules in HCC cells and liver cancer patient samples, excitingly, we have also found the regulatory relationship in tumour tissues from HCCLM3‐transplanted mice. There was a negative correlation between ETV6 and miR‐429 in vivo, ETV6 knockdown increased the expression level of miR‐429 in tumour tissues from HCCLM3‐transplanted mice (Figure 9A), meanwhile, there was also a negative correlation between miR‐429 and CRKL in vivo, miR‐429 overexpression decreased the protein expression level of CRKL (Figure 9B). ETV6‐miR‐429‐CRKL regulatory loop regulates the in vitro glucose metabolism of HCC cells via the PI3K/AKT pathway, whether this effect also plays through PI3K/AKT pathway in vivo, we surprise found ETV6/CRKL knockdown and miR‐429 overexpression decreased the protein expression levels of PI3K110, PI3K85, p‐AKT in tumour tissues from HCCLM3‐transplanted mice (Figure 9C). The above results demonstrate ETV6 binds to miR‐429 to mediate in vivo glucose metabolic reprogramming of HCC cells by targeting CRKL via PI3K/AKT pathway.

## 5 Conclusion

**FIGURE 10:**
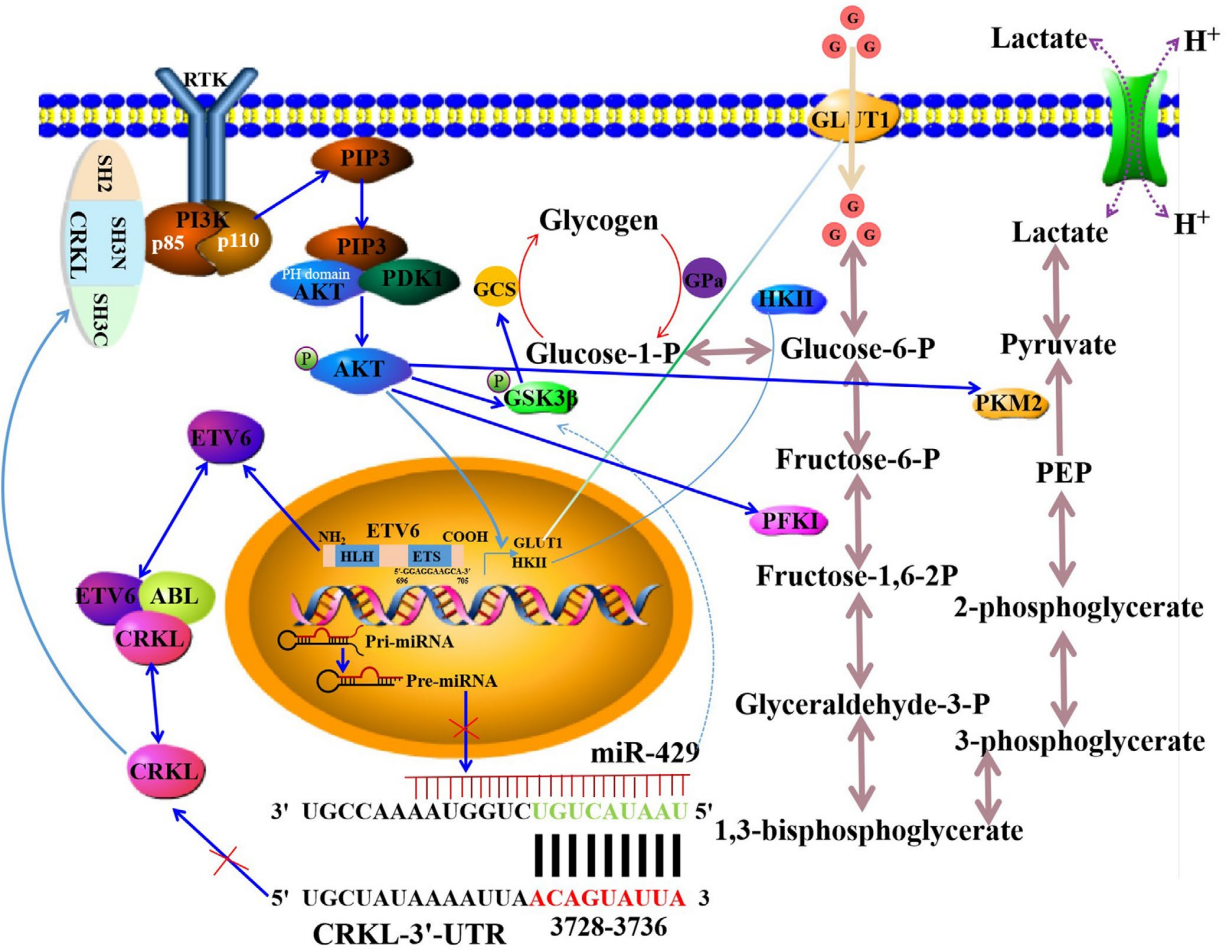
The underlying mechanism of ETV6‐miR‐429‐CRKL regulatory loop on glucose metabolism reprogramming of HCC. ETV6 binds to miR‐429 to mediate glucose metabolic reprogramming of HCC by targeting CRKL via the PI3K/AKT pathway by regulating aerobic glycolysis and glycogen synthesis‐related key molecules, miR‐429 couples to the aerobic glycolysis pathway by mediating the glycogen shunt.

In this study, we systematically investigated the role of ETV6 in the reprogramming of glucose metabolism and its potential mechanism in HCC cells, both in vitro and in vivo. Our findings reveal, for the first time, that the ETV6‐miR‐429‐CRKL regulatory loop controls glucose metabolic reprogramming in HCC cells; the proposed mechanism was summarised in Figure 10. The ETV6 ETS domain directly binds to the promoter region of miR‐429 to suppress its expression, then miR‐429 negatively regulates CRKL expression by targeting the 3′‐UTR of *CRKL*. Subsequently, the SH3N domain of CRKL directly binds to the p85 PxxP motif of PI3K, leading to PI3K activation and the production of PIP2 and PIP3 at the intracellular membrane. Then, PIP3 binds to AKT via its PH domain and PDK1 phosphorylates AKT at Thr308, resulting in AKT activation (p‐AKT). Subsequently, p‐AKT promotes the transcription and plasma membrane localization of GLUT1, enhancing glucose uptake by facilitating glucose transport into cells. Additionally, p‐AKT upregulates the transcription and activity of HKII, promoting the generation of G‐6‐P. G‐6‐P undergoes glycolysis, leading to the sequential conversion of F‐6‐P, F‐1,6–2P, PEP, and pyruvate into lactate by PFK1 and PKM2, which are activated by p‐AKT. Meanwhile, p‐AKT directly phosphorylates and inactivates GSK3β, preventing the phosphorylation and inactivation of GCS; this allows GCS to remain active and promote glycogen synthesis. Furthermore, miR‐429 modulates the rate of glycogen production and degradation via enhanced activities of GCS and GPa to promote glycogen synthesis, then coupling aerobic glycolytic pathways by mediating glycogen shunting.

Glucose metabolic reprogramming is an important hallmark of tumour cells, metabolic alterations in cancer cells provide avenues for the development of cancer cell‐specific therapeutic targets and anticancer drugs, the designed inhibitors target to tumour glucose metabolic reprogramming will become an effective treatment strategy for tumours. In the future, we hope the targeted‐ETV6‐miR‐429‐CRKL regulatory loop drug will be designed to effectively inhibit glucose metabolism, which provides fundamental sight of molecule‐targeted therapy of cancer, molecule‐targeted therapy will represent the future development direction of treatment of tumours. Current work demonstrates ETV6‐miR‐429‐CRKL‐PI3K/AKT regulatory pathway in glucose metabolism of HCC. Herein, our work highlights the clue to suppress ETV6‐miR‐429‐CRKL regulatory loop in inhibiting glucose metabolism of HCC cells, which provides a new fundamental sight and clue of cancer molecule‐targeted therapy. The innovations targeting reprogramming of glucose metabolism related will be the hotspot for the research of cancer therapy in the future.

Although conventional chemotherapy effectively targets tumour cells, its lack of specificity often leads to severe side effects. However, key differences between cancer and healthy cells—such as metabolic alterations—can be exploited to improve treatment precision. One such difference is the Warburg effect, a hallmark of cancer characterised by heightened glycolysis in tumour cells, enabling their survival in hypoxic and acidic conditions while promoting aggressiveness and chemoresistance. Elevated glycolysis not only acidifies the tumour microenvironment (TME), reducing drug stability and efficacy, but also generates biosynthetic intermediates that fuel proliferation and ATP‐dependent drug efflux mechanisms, contributing to multidrug resistance. Moreover, cancer cells exhibit metabolic plasticity, dynamically shifting between glycolysis and oxidative phosphorylation (OXPHOS) to adapt to TME conditions. Targeting these metabolic vulnerabilities could enable more precise and effective therapies. Yet, current treatments often overlook the dynamic interplay between glycolytic and oxidative phenotypes, a key driver of tumour heterogeneity. A deeper understanding of metabolic rewiring across cancer types and stages—along with TME factors like oxygen levels, pH, and nutrient availability—is critical for developing strategies to overcome resistance. Integrating metabolic insights with advanced delivery systems (e.g., nanoparticles) and combination therapies could revolutionise drug development, enhance specificity, and address the persistent challenge of treatment resistance in oncology [60].

## Author Contributions

**Chunmei Guo:** formal analysis, funding acquisition, investigation, supervision, validation, writing – original draft, writing – review and editing. **Lingqian Xie:** data curation, investigation, methodology, software, validation. **Huiqing Yin:** data curation, investigation, methodology, validation. **Lina Yi:** data curation, methodology, validation. **Lin Jin:** data curation, investigation, methodology. **Xiangwei Liu:** data curation, methodology. **Qingqing Zhang:** methodology. **Zijian Li:** software, visualisation. **Shuqing Liu:** supervision, resources, project administration, conceptualization. **Ming‐Zhong Sun:** supervision, resources, project administration, conceptualization, writing – review and editing. All authors read and approved the final manuscript.

## Ethics Statement

The Nude BABL/c mice were provided by the specific pathogen free (SPF) Animal Laboratory Center of Dalian Medical University, the mice were housed under standard conditions and treated in accordance with institutional guidelines for laboratory animal care. The study was conducted following the ethical standards of the national and international regulations and was approved by the Experiment Animal Ethical Committee of Dalian Medical University (Approval Number: AAE24056).

## Conflicts of Interest

The authors declare no conflicts of interest.

## Data Availability

The data that support the findings of this study are available on request from the corresponding author. The data are not publicly available due to privacy or ethical restrictions.
